# Space-time wave packets localized in all dimensions

**DOI:** 10.1038/s41467-022-32240-0

**Published:** 2022-08-05

**Authors:** Murat Yessenov, Justin Free, Zhaozhong Chen, Eric G. Johnson, Martin P. J. Lavery, Miguel A. Alonso, Ayman F. Abouraddy

**Affiliations:** 1 CREOL, The College of Optics & Photonics, University of Central Florida, Orlando, FL 32816 USA; 2 Micro-Photonics Laboratory, the Holcombe Department of Electrical and Computer Engineering, Clemson University, Clemson, SC 29634 USA; 3 James Watt School of Engineering, University of Glasgow, Glasgow, UK; 4 CNRS, Centrale Marseille, Institut Fresnel, Aix Marseille Univ., Marseille, France; 5 The Institute of Optics, University of Rochester, Rochester, NY USA

**Keywords:** Optical physics, Other photonics

## Abstract

Optical wave packets that are localized in space and time, but nevertheless overcome diffraction and travel rigidly in free space, are a long sought-after field structure with applications ranging from microscopy and remote sensing, to nonlinear and quantum optics. However, synthesizing such wave packets requires introducing non-differentiable angular dispersion with high spectral precision in two transverse dimensions, a capability that has eluded optics to date. Here, we describe an experimental strategy capable of sculpting the spatio-temporal spectrum of a generic pulsed beam by introducing arbitrary radial chirp via two-dimensional conformal coordinate transformations of the spectrally resolved field. This procedure yields propagation-invariant ‘space-time’ wave packets localized in all dimensions, with tunable group velocity in the range from 0.7*c* to 1.8*c* in free space, and endowed with prescribed orbital angular momentum. By providing unprecedented flexibility in sculpting the three-dimensional structure of pulsed optical fields, our experimental strategy promises to be a versatile platform for the emerging enterprise of space-time optics.

## Abstract (web summary)

Propagation-invariant wave packets confined in space and time can be useful for optical sensing, imaging, and nonlinear and quantum optics. Here the authors demonstrate control over the angular dispersion of optical wave packets in two-transverse dimensions to synthesize space-time wave packets localized in all dimensions.

## Introduction

Creating spatio-temporally localized optical wave packets that overcome diffraction and propagate rigidly in free space has been a long-standing yet elusive goal in optics. Such wave packets can have applications ranging from remote optical sensing and biological imaging, to nonlinear and quantum optics. To date, this challenge has been addressed via nonlinear optical effects that sustain solitons^1^, waveguiding structures^2^, or by exploiting particularly shaped waveforms such as Bessel-Airy wave packets in linear dispersive media^3^. Propagation invariance in a linear nondispersive medium necessitates inculcating a precise spatio-temporal spectral structure into the field by introducing angular dispersion (AD)^4,5^; i.e., associating each wavelength with a single propagation direction^6,7^. Examples of such wave packets date back to Brittingham’s focus-wave mode^8^, X-waves^9,10^, and more recently the general class of ‘space-time’ (ST) wave packets^11–19^. The challenge of producing the AD necessary for propagation-invariant wave packets localized in all dimensions (referred to hereon as 3D ST wave packets) is twofold. First, the AD must be inculcated in two transverse dimensions rather than in one as typically realized via gratings or prisms^4,5^. Second, non-differentiable AD is required^20^; i.e., it is necessary that the derivative of the wavelength-dependent propagation angle not be defined at some wavelength^21,22^ – a field configuration that cannot be directly produced with conventional optical components. Consequently, with the exception of X-waves that are AD-free, no propagation-invariant optical wave packets that are localized in all dimensions have been observed in free space^7^.

The challenge of introducing arbitrary AD into a generic pulsed beam along one transverse dimension has been recently addressed by constructing a universal AD synthesizer^23^. This experimental strategy has enabled the realization of ST wave packets in the form of light sheets^16^ (referred to hereon as 2D ST wave packets), which exhibit a broad host of sought-after effects, such as long-distance propagation invariance^24^, tunable group velocities^13,25–29^, anomalous refraction at planar interfaces^30^, and the space-time Talbot effect^31^. Although this arrangement produces non-differentiable AD with high spectral resolution, these features cannot be extended to both transverse dimensions. Crucially, the centerpiece of this configuration is a spatial light modulator that modifies the temporal spectrum along one dimension, leaving only one dimension to manipulate the field spatially – a limitation that is shared by other recently investigated spatio-temporal field structures^32–39^. Therefore, the fundamental challenge of producing non-differentiable AD encompassing both transverse dimensions remains outstanding.

Here, we demonstrate a spatio-temporal modulation strategy that efficiently produces arbitrary yet precise AD in two transverse dimensions, and thus yields ST wave packets localized in all dimensions – while preserving all the key attributes of its reduced-dimension counterpart. This modulation scheme is implemented in three stages. In the first stage, the spectrum of a generic plane-wave pulse is spatially resolved along one dimension after a double-pass through a volume chirped Bragg grating. In the second stage, a spectral transformation ‘reshuffles’ the wavelengths into a prescribed sequence. In the third stage, a log-polar-to-Cartesian conformal coordinate transformation converts the spatial locus of each wavelength from a line into a circle^40,41^. A lens finally converts the spectrally resolved wave front into a 3D ST wave packet localized in all three dimensions. Utilizing this approach, we produce 3D ST wave packets with ≈30 μm transverse beam width and ≈6 ps pulse width that propagate for over 50 mm. Moreover, by modulating the spatio-temporal spectral structure, we realize group velocities extending from the subluminal to the superluminal regimes over the range from 0.7*c* to 1.8*c* (*c* is the speed of light in vacuum). Furthermore, by providing access to both transverse dimensions in a ST wave packet, new degrees of freedom of the optical field can be accessed, such as orbital angular momentum (OAM)^42–44^. Specifically, by encoding a helical phase structure in the spatio-temporal spectrum, we demonstrate propagation-invariant pulsed OAM wave packets with controllable group velocity in free space, which we refer to as ST-OAM wave packets. In addition to the propagation-invariance and arbitrary group velocities of ST-OAM wave packets, their underlying spatio-temporal structure may lead to variations of some of the recently uncovered behaviors of conventional OAM pulses, such as the trade-off between the topological charge and pulse duration^43,45,46^. Such 3D ST wave packets that are fully localized in all dimensions have potential uses in areas such as free-space optical communications, imaging, and nonlinear optics.

## Results

### Theory of 3D space-time wave packets

**Fig. 1:**
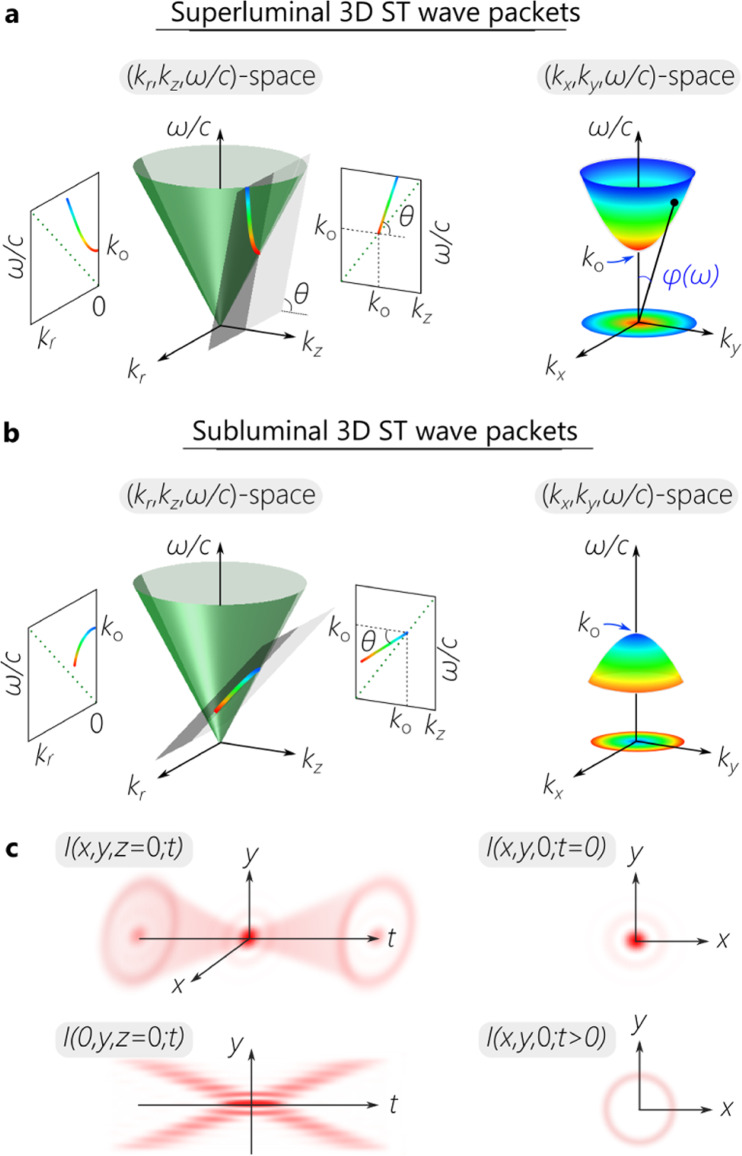
Visualization of the spectral support domain for 3D ST wave packets on the surface of the free-space light-cone. **a** The spectral support domain for a superluminal 3D ST wave packet at the intersection of the light-cone ${k}_{r}^{2}+{k}_{z}^{2}\,=\,{(\frac{\omega }{c})}^{2}$ with a spectral plane that is parallel to the *k*_*r*_-axis and makes an angle *θ* > 45^∘^ with the *k*_*z*_-axis. The conic section at the intersection is a hyperbola. In $({k}_{x},{k}_{y},\frac{\omega }{c})$-space the spectrum is one half of a two-sheet hyperboloid (an elliptic hyperboloid). **b** Same as **a** for a subluminal ST wave packet with *θ* < 45^∘^, where the spectral support domain on the light-cone in $({k}_{r},{k}_{z},\frac{\omega }{c})$-space is an ellipse. In $({k}_{x},{k}_{y},\frac{\omega }{c})$-space, the spectrum is an ellipsoid of revolution (a spheroid, which may be prolate or oblate according to the value of *θ*). **c** Plot of the spatio-temporal intensity profile *I*(*x*, *y*, *z* = 0; *t*) at a fixed axial plane *z* = 0, the intensity profile in a meridional plane *I*(0, *y*, *z* = 0; *t*), and the transverse profiles at the wave-packet center *I*(*x*, *y*, 0; 0) and off-center *I*(*x*, *y*, 0; *t* > 0).

A useful conceptual tool for understanding the characteristics of ST wave packets and the requirements for their synthesis is to visualize their spectral support domain on the surface of the light cone. The light-cone is the geometric representation of the free-space dispersion relationship ${k}_{x}^{2}+{k}_{y}^{2}+{k}_{z}^{2}\,=\,{(\frac{\omega }{c})}^{2}$, where *ω* is the temporal frequency, *c* is the speed of light in vacuum, (*k*_*x*_, *k*_*y*_, *k*_*z*_) are the components of the wave vector in the Cartesian coordinate system (*x*, *y*, *z*), *x* and *y* are the transverse coordinate, and *z* is the axial coordinate. Although this relationship corresponds to the surface of a four-dimensional hypercone, a useful representation follows from initially restricting our attention to azimuthally symmetric fields in which *k*_*x*_ and *k*_*y*_ are combined into a radial wave number ${k}_{r}\,=\,\sqrt{{k}_{x}^{2}+{k}_{y}^{2}}$, so that the light-cone can be then visualized in $({k}_{r},{k}_{z},\frac{\omega }{c})$-space (Fig. 1). The spectral support domain for 3D ST wave packets is restricted to the conic section at the intersection of the light-cone with a spectral plane that is parallel to the *k*_*r*_-axis and makes an angle *θ* (the spectral tilt angle) with the *k*_*z*_-axis, which is given by the equation ${{\Omega }}\,=\,({k}_{z}-{k}_{{{{{{{{\rm{o}}}}}}}}})c\tan \theta$; here Ω = *ω* − *ω*_o_, *ω*_o_ is a carrier frequency, and *k*_o_ = *ω*_o_/*c*. It can be readily shown that such a construction in the narrowband paraxial regime results in a propagation-invariant 3D ST wave packet $E(r,\, z;t)\,=\,{e}^{i({k}_{{{{{{{{\rm{o}}}}}}}}}z-{\omega }_{{{{{{{{\rm{o}}}}}}}}}t)}\psi (r,\, z;t)$, where the slowly varying envelope *ψ*(*r*, *z*; *t*) travels rigidly at a group velocity $\widetilde{v}\,=\,c\tan \theta$, $\psi (r,z;t)\,=\,\psi (r,0;t-z/\widetilde{v})$, where $\psi (r,0;t)\,=\,\int \,d{k}_{r}\,\,{k}_{r}\widetilde{\psi }({k}_{r}){J}_{0}({k}_{r}r){e}^{-i{{\Omega }}t}$, and $\widetilde{\psi }({k}_{r})$ is the spectrum. Here *k*_*r*_ and Ω are no longer independent variables, but are instead related via the particular spectral trajectory on the light-cone (Supplementary Note 1). Although this spectral trajectory is a conic section whose kind is determined by the spectral tilt angle *θ*, it can nevertheless be approximated in the narrowband paraxial regime by a parabola in the vicinity of *k*_*r*_ = 0:

1 $$\frac{{{\Omega }}}{{\omega }_{{{{{{{{\rm{o}}}}}}}}}}=\frac{{k}_{r}^{2}}{2{k}_{{{{{{{{\rm{o}}}}}}}}}^{2}(1-\widetilde{n})},$$

where $\widetilde{n}\,=\,\cot \theta$ is the wave-packet group index in free space. By setting ${k}_{r}\,=\,k\sin \varphi (\omega )$, where *φ*(*ω*) is the propagation angle for *ω* as shown in Fig. 1a, we have $\varphi (\omega )\,\approx \,\eta \sqrt{\frac{{{\Omega }}}{{\omega }_{{{{{{{{\rm{o}}}}}}}}}}}$, which is not differentiable at Ω = 0^20,23^; here $\widetilde{n}\,=\,1-\frac{\sigma }{2}{\eta }^{2}$, *σ* = 1 in the superluminal regime, and *σ* = − 1 in the subluminal regime. In other words, non-differentiable AD is required to produce a propagation-invariant ST wave packet. This result is similar to that for ST light-sheets^16^ except that the transverse coordinate *x* is now replaced with the radial coordinate *r*.

The representation in Fig. 1 is particularly useful in identifying a path towards synthesizing 3D ST wave packets. When 45° < *θ* < 90°, the ST wave packet is superluminal $\widetilde{v}\, > \,c$, Ω is positive, and *ω*_o_ is the minimum allowable frequency in the spectrum. When viewed in $({k}_{x},{k}_{y},\frac{\omega }{c})$-space, the wavelengths are arranged in concentric circles, with long wavelengths (low frequencies) at the center, and shorter wavelengths (higher frequencies) extending outward. On the other hand, when 0° < *θ* < 45°, the ST wave packet is subluminal $\widetilde{v}\, < \,c$, Ω is negative, and *ω*_o_ is the maximum allowable frequency in the spectrum. The wavelengths are again arranged in concentric circles in $({k}_{x},{k}_{y},\frac{\omega }{c})$-space – but in the opposite order: short wavelengths are close to the center and longer wavelengths extend outward. For both subluminal and superluminal 3D ST wave packets, each *ω* is associated with a single radial spatial frequency *k*_*r*_(*ω*), and is related to it via the relationship in Eq. (1). This representation indicates the need for arranging the wavelengths in concentric circles with square-root radial chirp, and then converting the spatial spectrum into physical space via a spherical lens. Moreover, adding a spectral phase factor *e*^*iℓχ*^, where *ℓ* is an integer and *χ* is the azimuthal angle in spectral space, produces OAM in physical space (Supplementary Note 1B).

Closed-form expressions can be obtained for 3D ST wave packets by applying Lorentz boosts to an appropriate initial field^47–50^. For example, starting with a monochromatic beam *E*_o_(*r*, *z*; *t*), a subluminal 3D ST wave packet at a group velocity $\widetilde{v}$ is obtained by the Lorentz boost $E(r,\, z;t)\,=\,{E}_{{{{{{{{\rm{o}}}}}}}}}(r,\frac{z-\widetilde{v}t}{\sqrt{1-{\beta }^{2}}};\frac{t-\widetilde{v}z/{c}^{2}}{\sqrt{1-{\beta }^{2}}})$, where $\beta \,=\,\frac{\widetilde{v}}{c}$ is the Lorentz factor. On the other hand, closed-form expressions for superluminal 3D ST wave packets can be obtained by applying a Lorentz boost to the ‘needle beam’ in^12^. The time-averaged intensity is $I(r,\varphi,z)\,=\,2\pi {k}_{{{{{{{{\rm{o}}}}}}}}}^{2}(1-\widetilde{n})\int d{k}_{r}{k}_{r}^{2}|\widetilde{\psi }({k}_{r}){|}^{2}{J}_{\ell }^{2}({k}_{r}r)$, which is independent of *φ* even if the field is endowed with OAM. In the case of 2D ST light-sheets, the time-averaged intensity separates into a sum of a constant background pedestal and a spatially localized feature at the center^16^. A similar decomposition is not possible for 3D ST wave packets. However, using the asymptotic form for Bessel functions that is valid far from *r* = 0, we have:

2 $$I(r)\,\approx\, 	\frac{\,2\pi {k}_{{{{{{{{\rm{0}}}}}}}}}^{2}(1-\widetilde{n})}{\pi r}\int \,d{k}_{r}\sqrt{{k}_{{{{{{{{\rm{o}}}}}}}}}^{2}+{k}_{r}^{2}}|\widetilde{\psi }({k}_{r}){|}^{2} \\ 	+\frac{\,2\pi {k}_{{{{{{{{\rm{o}}}}}}}}}^{2}(1-\widetilde{n}){(-1)}^{\ell }}{\pi r}\int \,d{k}_{r}\sqrt{{k}_{{{{{{{{\rm{o}}}}}}}}}^{2}+{k}_{r}^{2}}|\widetilde{\psi }({k}_{r}){|}^{2}\sin (2{k}_{r}r),$$

where the first term is a pedestal decaying at a rate of $\frac{1}{r}$, and the second term tends to be localized closer to the beam center. In the vicinity of *r* = 0, the two terms merge and cannot be separated. The spatio-temporal intensity profile of such a 3D ST wave packet is depicted in Fig. 1c: two conic field structures emanate from the wave-packet center, such that the profile is X-shaped in any (meridional plane containing the optical axis, and the intensity profile is circularly symmetric in any transverse plane.

### Synthesizing ST wave packets localized in all dimensions

**Fig. 2:**
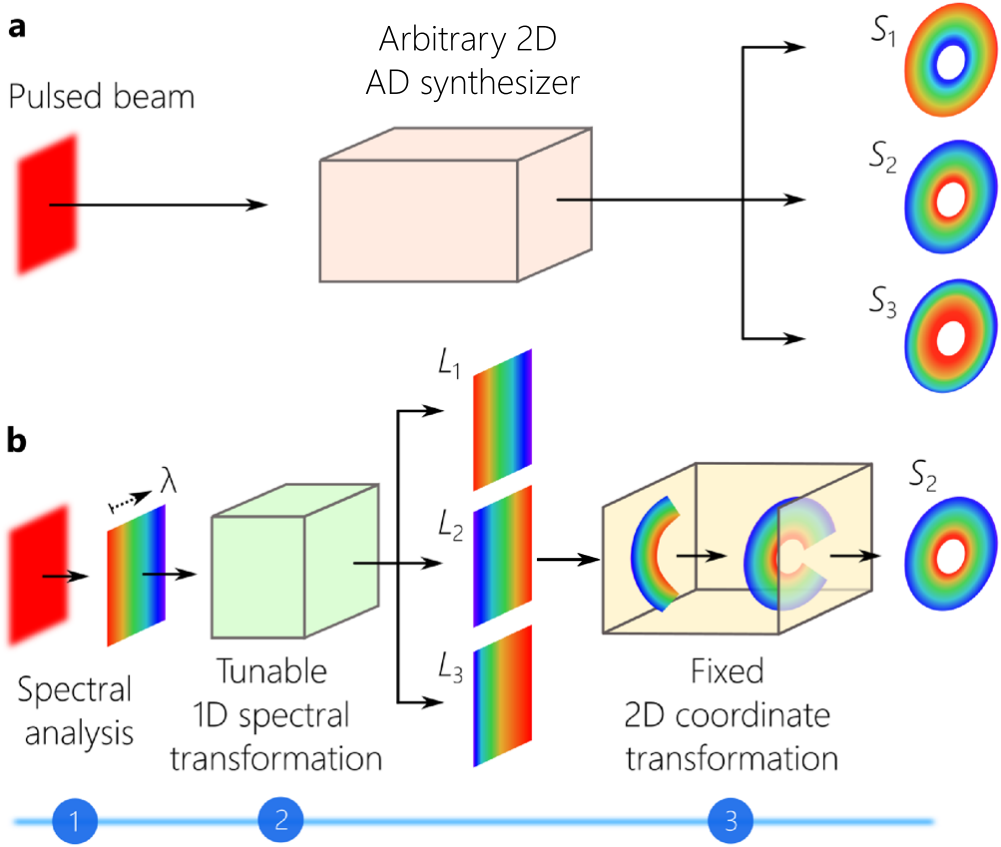
Synthesis strategy for 3D ST wave packets. **a** Starting with a generic plane-wave pulse, we aim at constructing an angular-dispersion synthesizer in two dimensions that arranges the wavelengths in circles in a prescribed order. *S*_1_ corresponds to a subluminal wave packet, whereas *S*_2_ and *S*_3_ correspond to superluminal wave packets of different group velocities. **b** The proposed strategy comprises spectral analysis followed by a tunable 1D spectral transformation that rearranges the initial wavelength sequence in the spectrally resolved wavefront. The 1D spectra *L*_1_, *L*_2_, and *L*_3_ are rectilinear counterparts of *S*_1_, *S*_2_, and *S*_3_ in **a**. In the third stage, a fixed 2D conformal coordinate transformation converts vertical lines into circles, thereby realizing the targeted spatio-temporal spectra *S*_1_, *S*_2_, and *S*_3_.

Central to converting a generic pulsed beam into a ST wave packet localized in all dimensions is the construction of an optical scheme that can associate each wavelength *λ* with a particular azimuthally symmetric spatial frequency *k*_*r*_(*λ*) and arrange the wavelengths in concentric circles with the order prescribed in Eq. (1) (Fig. 2a). This system realizes two functionalities, producing a particular wavelength sequence, and changing the coordinate system, which are implemented in succession via the three-stage strategy outlined in Fig. 2b. In the first stage, the spectrum of a plane-wave pulse is resolved along one spatial dimension. At this point, the field is endowed with linear spatial chirp and the wavelengths are arranged in a fixed sequence. The second stage rearranges the wavelengths in a new prescribed sequence. This spectral transformation is tunable; that is, a wide range of spectral structures can be obtained from a fixed input. In the third stage, a 2D conformal transformation converts the coordinate system to map the rectilinear chirp into a radial chirp; i.e., lines corresponding to different wavelengths at the input are converted into circles at the output^40,41^. Because the spectral transformation in the second stage is tunable, the 2D coordinate transformation can be held fixed. In this way, we obtain arbitrary (including non-differentiable) AD in two dimensions.

**Fig. 3:**
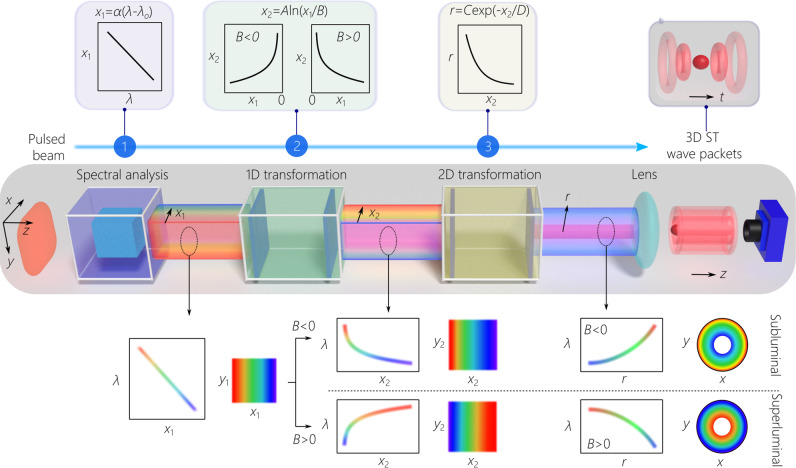
Schematic of the setup for synthesizing 3D ST wave packets. Starting with a plane-wave pulse on the left, spectral analysis resolves the spectrum in space and produces linear spatial chirp, *x*_1_(*λ*) = *α*(*λ* − *λ*_o_). The spectrally resolved field enters a tunable 1D spectral transformation formed of two spatial light modulators `reshuffles' the wavelengths, ${x}_{2}(\lambda )\,=\,A\ln (\frac{{x}_{1}(\lambda )}{B})$. Opposite signs of chirp along *x*_2_ are required for subluminal and superluminal wave packets. Next, a fixed 2D coordinate transformation (implemented with two fixed phase plates) converts the vertical lines corresponding to different wavelengths into circles of radius $r(\lambda )\,=\,C\exp (-\frac{{x}_{2}(\lambda )}{D})$. Finally, a converging spherical lens produces the 3D ST wave packet. On the top, we plot the implemented spectral and spatial transformations; on the bottom, we illustrate the field structure at different points along the setup.

The layout of the experimental setup is depicted in Fig. 3. We start off in the first stage with pulses from a Ti:sapphire laser (pulse width ≈ 100 fs and bandwidth ≈10 nm at a central wavelength of ≈800 nm). Because a flat-phase front is critical for successfully implementing the subsequent transformations, the use of conventional surface gratings is precluded, and we utilize instead a double-pass configuration through a volume chirped Bragg grating (CBG). The CBG resolves the spectrum horizontally along the *x*-axis and introduces linear spatial chirp so that *x*_1_(*λ*) = *α*(*λ* − *λ*_o_); where *α* is the linear spatial chirp rate^51^, *λ*_o_ is a fixed wavelength, and the bandwidth utilized is Δ*λ* ≈ 0.3 nm. It is crucial that this task be achieved with high spectral resolution. Previous studies have shown that the critical parameter determining the propagation distance of ST wave packets is the ’spectral uncertainty’ *δλ*, which is the finite spectral uncertainty in the association between spatial and temporal frequencies^52^. Our measurements indicate that the optimal spectral uncertainty after the CBG arrangement is *δλ* ~ 35 pm, which is achieved for a 2-mm input beam width (Supplementary Fig. 11).

The second stage of the synthesis strategy is a 1D spatial transformation along the *x*-axis to rearrange the wavelength sequence, thereby implementing a spectral transformation. Specifically, each wavelength *λ* is transposed from *x*_1_(*λ*) at the input via a logarithmic mapping to ${x}_{2}(\lambda )\,=\,A\ln (\frac{{x}_{1}(\lambda )}{B})$ at the output. This transformation is realized via two phase patterns implemented by a pair of spatial light modulators (SLMs) to enable tuning the transformation parameters *A* and *B*. This particular ‘reshuffling’ of the wavelength sequence pre-compensates the exponentiation included in the subsequent coordinate transformation. By tuning the value of *B*, we can vary the group velocity $\widetilde{v}$ over the subluminal and superluminal regimes (Supplementary Table 1).

In the third stage we perform a log-polar-to-Cartesian coordinate transformation: (*x*_2_, *y*_2_) → (*r*, *φ*) via the 2D mapping: $r(\lambda )\,=\,C\exp (-\frac{{x}_{2}(\lambda )}{D})$ and $\varphi=\frac{{y}_{2}}{D}$^40,41^. The exponentiation here is pre-compensated by the logarithmic mapping in the 1D spectral transformation, and the wavelength at position *x*_2_(*λ*) at the input is converted into a circle of radius $r(\lambda )\,\propto \,{(\lambda -{\lambda }_{{{{{{{{\rm{o}}}}}}}}})}^{A/D}$ at the output. This 2D coordinate transformation was developed decades ago^40,41^, and was recently revived as a methodology for sorting OAM modes^53,54^. We operate the system in reverse (lines-to-circles, rather than the more typical circles-to-lines^53^), and we make use of a polychromatic field (rather than monochromatic field). The exponent of the chirp rate depends only on the ratio $\frac{A}{D}$, so that setting *D* = 2*A* yields $r(\lambda )\,\propto \,\sqrt{\lambda -{\lambda }_{{{{{{{{\rm{o}}}}}}}}}}$ in accordance with Eq. (1). The wavelengths are arranged with square-root radial chirp, thereby realizing the required non-differentiable AD. Finally, a spherical converging lens of focal length *f* generates the 3D ST wave packets in physical space, equivalently mapping $r\to \,{k}_{r}\,=\,k\frac{r}{f}$.

**Fig. 4:**
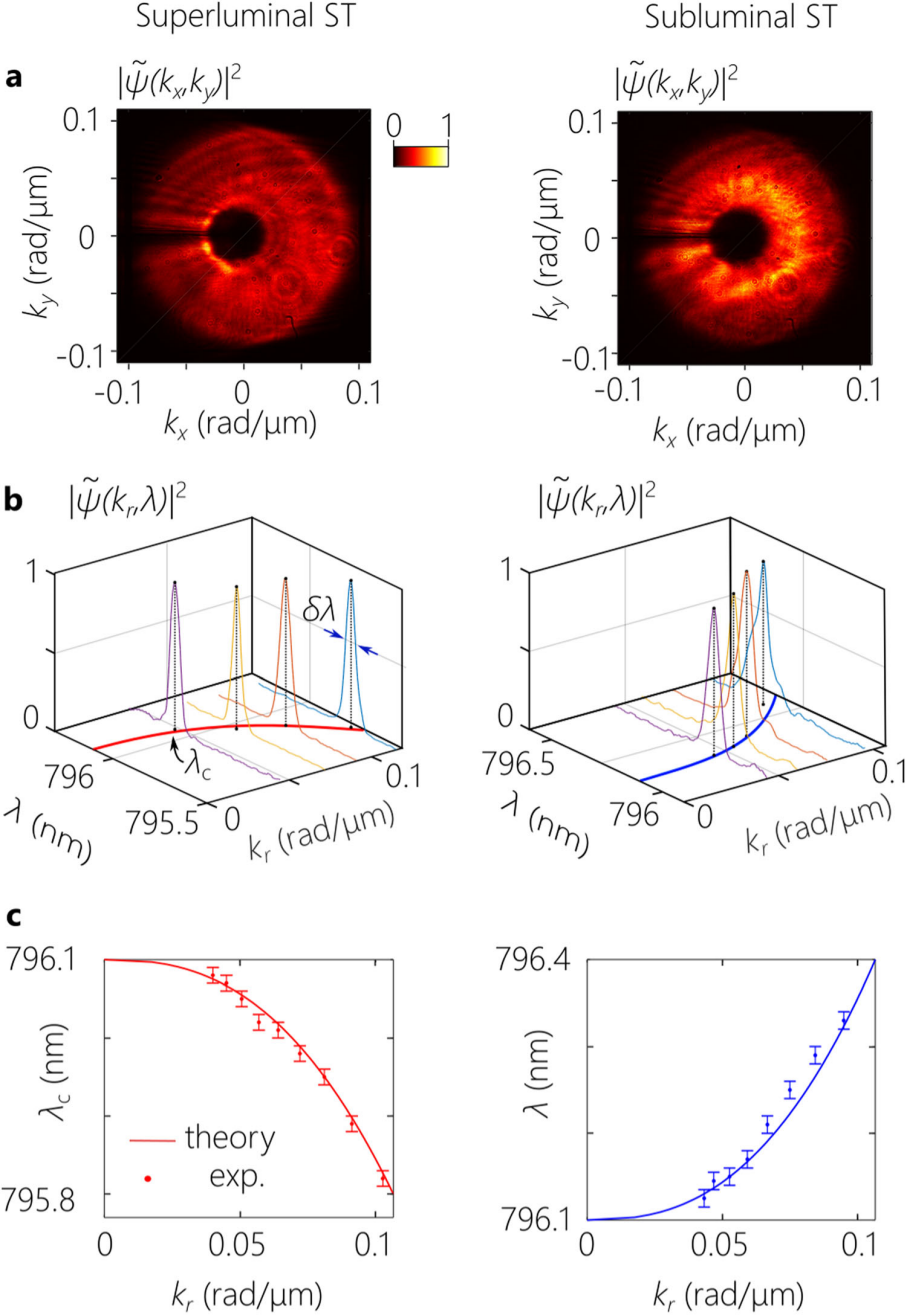
The spatio-temporal spectral structure of 3D ST wave packets. Measurements for a superluminal wave packet ($\widetilde{v}\,\approx \,1.37c$) are plotted in the left column, and those for its subluminal counterpart ($\widetilde{v}\,\approx \,0.83c$) are plotted in the column on the right. **a** Measured spatial spectrum $|\widetilde{\psi }({k}_{x},{k}_{y},\lambda ){|}^{2}$ by a wavelength-insensitive camera showing an annular structure. **b** Measured temporal spectra at selected radial positions revealing the radial chirp and the spectral uncertainty. **c** Measured radial chirp by plotting the central wavelength *λ*_c_ of the spectrum with radial spatial frequency *k*_*r*_. Error bars in **c** represent the spectral resolution of the optical spectrum analyzer (OSA; Advantest AQ6317B) we made use to perform spectral measurements.

The 2D coordinate transformation is performed with two different embodiments: using a pair of diamond-machined refractive phase plates^54^, and using a pair of diffractive phase plates^55^, which yielded similar performance. Because both of these realizations are stationary, the values of *C* and *D* are fixed. The data reported in Fig. 4 through Fig. 7 made use of the refractive phase plates with *C* = 4.77 mm and *D* = 1 mm. Moreover, fixing the value of *D* entails in turn fixing the value of *A* to maintain *A* = *D*/2. The group velocity $\widetilde{v}\,=\,c/\widetilde{n}$ is tuned over the subluminal and superluminal regimes by varying *B*, whereby $\widetilde{n}\,\approx \,1-\frac{2.24}{B}$, with *B* in units of mm [Supplementary Note 2].

This experimental strategy provides two pathways for introducing OAM into the 3D ST wave packet. One may utilize a conventional spiral phase plate to imprint an OAM order *ℓ* after the 2D coordinate transformation and before the final Fourier-transforming lens. Another approach, which we implemented here, is to add at the output of the 1D spectral transformation a linear phase distribution along *y* extending from 0 to 2πℓ, which is subsequently wrapped around the azimuthal direction after traversing the 2D coordinate transformation, thereby realizing OAM of order *ℓ*^55^.

For the sake of benchmarking, we also synthesized pulsed Bessel beams with separable spatio-temporal spectrum by circumventing the spectral analysis and 1D spectral transformation, and sending the input laser pulses directly to the 2D coordinate transformation. To match the temporal bandwidth of the pulsed Bessel beams to that of the 3D ST wave packets, we spectrally filter Δ*λ* = 0.3 nm from the input spectrum via a planar Fabry-Pérot cavity.

### Characterizing 3D ST wave packets

**Fig. 5:**
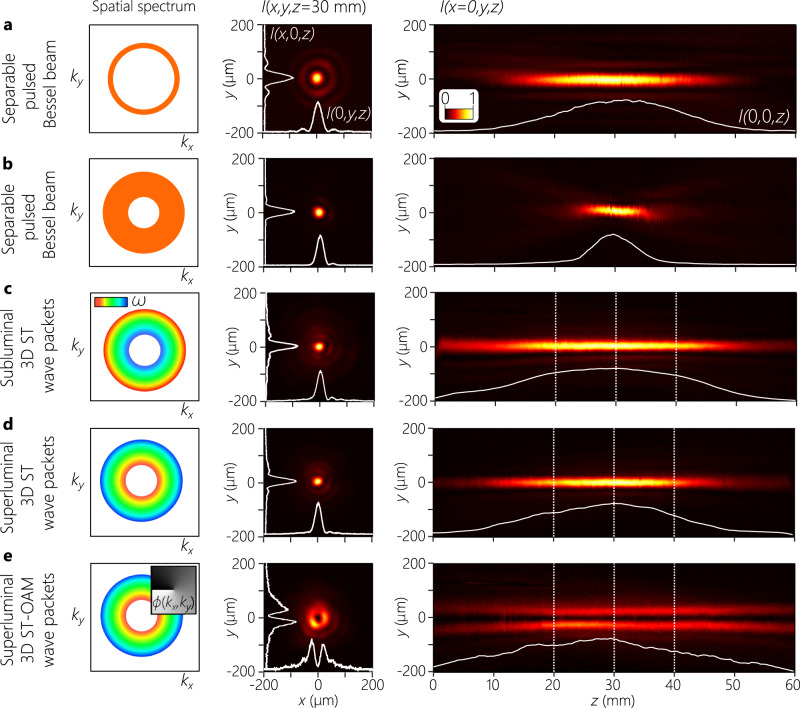
Measured transverse and axial time-averaged intensity for separable pulsed Bessel beams and 3D ST wave packets. In the first column, we illustrate the spatio-temporal structure; in the second, we plot the measured transverse intensity *I*(*x*, *y*, *z*) at *z* = 30 mm, in addition to sections through *x* = 0 and *y* = 0 (white curves); and, in the third, we plot the measured intensity in a meridional plane *I*(0, *y*, *z*). The white curve at the bottom of the panels in the last column is the on-axis intensity *I*(0, 0, *z*), except in **e** where we use *y* = 30 *μ*m. For all cases, Δ*λ* = 0.3 nm. **a** A separable pulsed Bessel beam with Δ*k*_*r*_ = 0.02 rad/*μ*m. **b** A pulsed Bessel beam with Δ*k*_*r*_ = 0.07 rad/*μ*m. **c**–**e** In all cases Δ*k*_*r*_ = 0.07 rad/*μ*m as in **b**. **c** A subluminal ($\widetilde{v}\,=\,0.83c$) 3D ST wave packet; **d** a superluminal ($\widetilde{v}\,=\,1.37c$) 3D ST wave packet; and **e** a superluminal ($\widetilde{v}\,=\,1.16c$) 3D ST-OAM wave packet with *ℓ* = 1 (the inset in the first column is the associated transverse spectral phase distribution). The dotted vertical white lines in the third column in **c-e** identify the axial planes for the time-resolved measurements in Fig. 6.

**Fig. 6:**
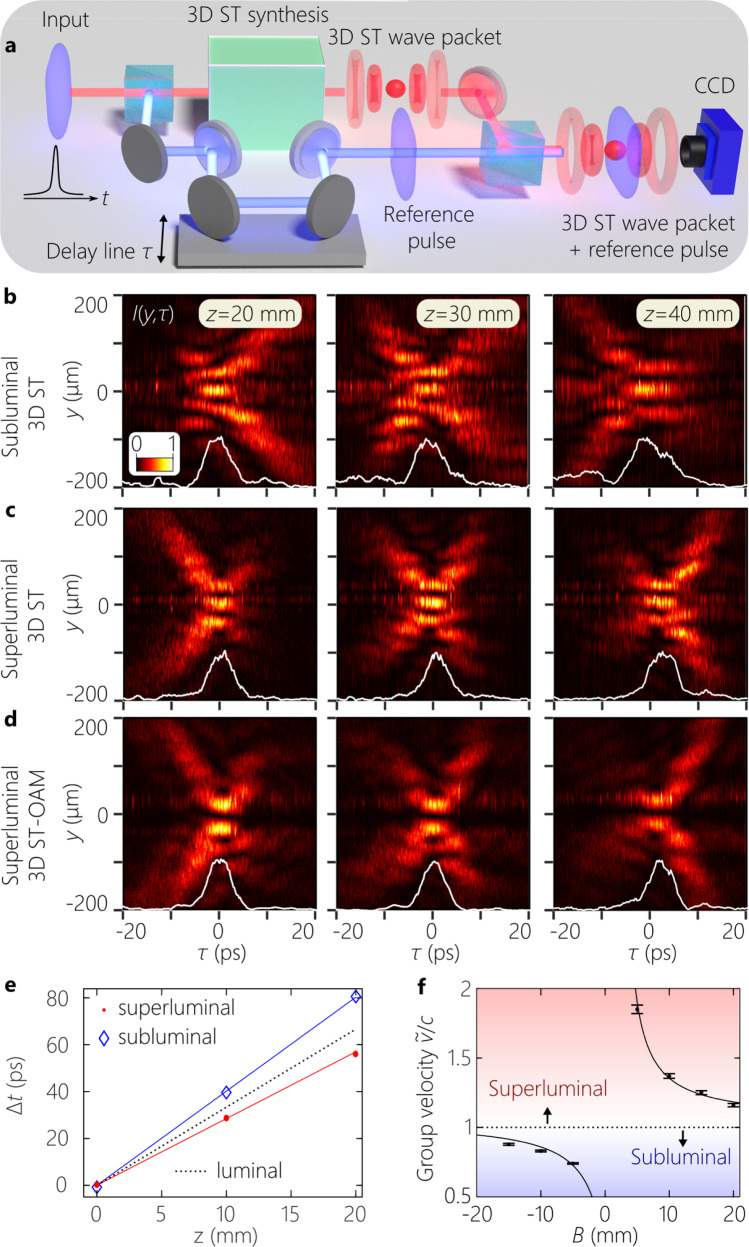
Reconstructing the spatio-temporal intensity profile and estimating the group velocity for 3D ST wave packets. **a** Schematic of the interferometric configuration for reconstructing *I*(*x*, *y*, *z*; *t*) and estimating $\widetilde{v}$. **b** Measured *I*(0, *y*, *z*; *τ*) at *z* = 20, 30, and 40 mm for a subluminal ($\widetilde{v}\,=\,0.83c$) wave packet; **c** for a superluminal ($\widetilde{v}\,=\,1.37c$) wave packet; and **d** for a superluminal ($\widetilde{v}\,=\,1.16c$) wave packet endowed with the OAM mode *ℓ* = 1. We also plot the section *y* = 0 through the intensity profile (white curve at the bottom of each panel), except in **d** where we use *y* = 30 *μ*m. **e** Measured group delay Δ*t* at different axial planes for subluminal and superluminal 3D ST wave packets. The straight lines are theoretical expectations and the symbols are data points. **f** Plot of the estimated group velocity $\widetilde{v}$ with the 1D spectral transformation parameter *B*. The curve is the theoretical expectation $\widetilde{v}\,=\,c/\widetilde{n}$, with $\widetilde{n}\,\approx \,1-\frac{2.24}{{{{{{{{\rm{B}}}}}}}}}$ (*B* in mm). Error bars correspond to the uncertainty in the measurement of $\widetilde{v}$ due to the finite pulse width of 3D ST wave packets; see Supplementary Note 3C.

**Fig. 7:**
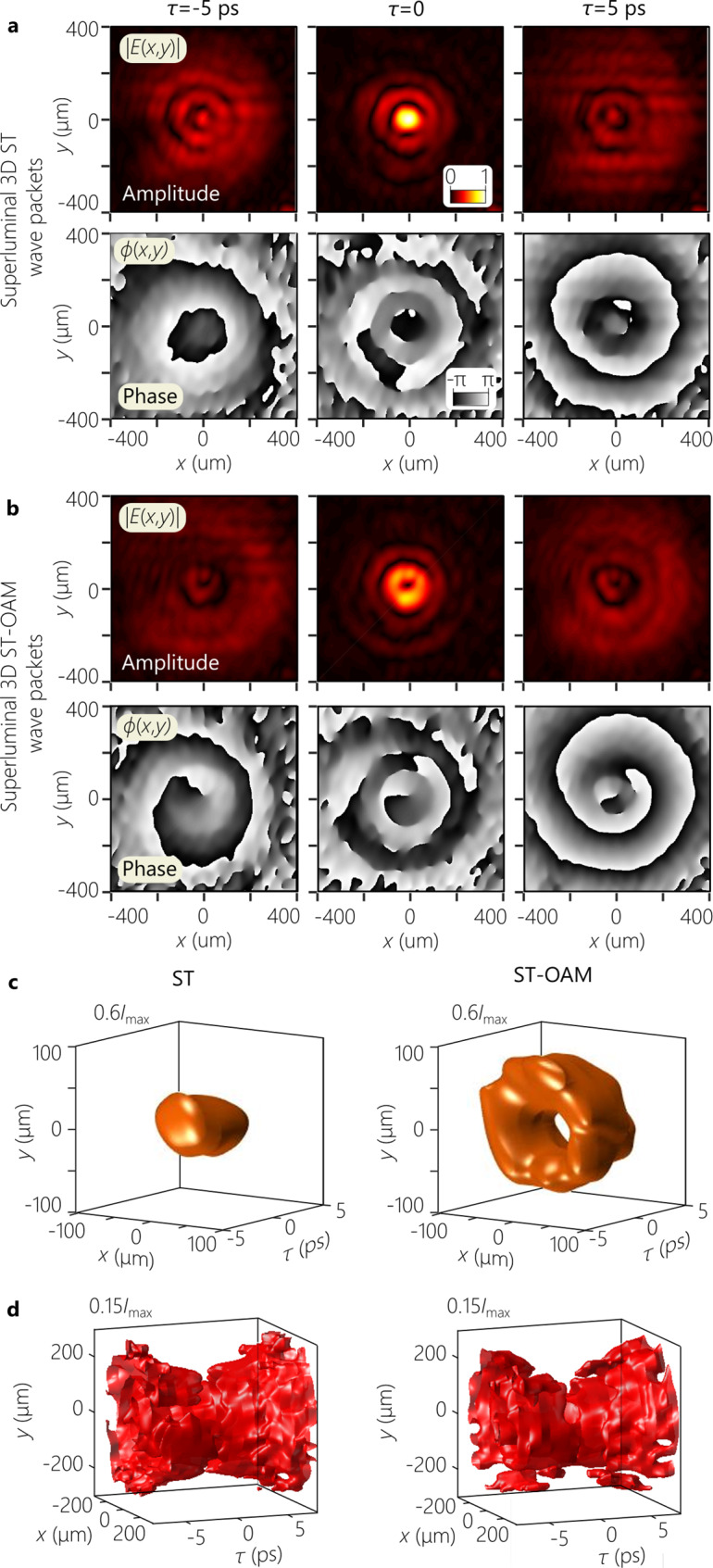
Measured complex-field amplitude and phase profiles for 3D ST wave packets with and without OAM. **a** Measured amplitude ∣*ψ*(*x*, *y*)∣ (first row) and phase *ϕ*(*x*, *y*) (second row) at a fixed axial plane *z* = 30 mm (see Figs. 5 and 6) at delays *τ* = −5 ps, *τ* = 0 corresponding to the wave-packet center, and *τ* = 5 ps for a superluminal 3D ST wave packet with *ℓ* = 0 and **a** *ℓ* = 1. **c** Iso-amplitude contour $I=0.6{I}_{\max }$ for the 3D ST wave packet from (**a**) and ST-OAM from (**b**). **d** Same as **c** but for the iso-amplitude contour $I=0.15{I}_{\max }$.

To verify the structure of the synthesized 3D ST wave packet, we characterize the field in four distinct domains: (1) the spatio-temporal spectrum to verify the square-root radial chirp (Fig. 4]; (2) the time-averaged intensity to confirm diffraction-free propagation along *z* (Fig. 5); (3) time-resolved intensity measurements to reconstruct the wave-packet spatio-temporal profile and estimate the group velocity (Fig. 6); and (4) complex-field measurements to resolve the spiral phase of the ST-OAM wave packets (Fig. 7).

#### Spectral-domain characterization

We measure the spatio-temporal spectrum by scanning a single-mode fiber connected to an optical spectrum analyzer across the spectrally resolved field profile. We scan the fiber along *x*_1_ after the spectral analysis stage and verify the linear spatial chirp (Supplementary Fig. 10), and then scan the fiber along *x*_2_ after the 1D spectral transformation to confirm the implemented change in spatial chirp. The measurement is repeated for superluminal (*B* = 10 mm, $\widetilde{v}\,\approx \,1.37c$) and subluminal (*B* = −10 mm, $\widetilde{v}\,\approx \,0.83c$) wave packets, both with temporal bandwidth Δ*λ* ≈ 0.3 nm, pulse width of ~ 6 ps, and *λ*_o_ = 796.1 nm. After the 2D coordinate transformation, the spectrum is arranged radially along an annulus rather than a rectilinear domain, as shown in Fig. 4a. By calibrating the conversion *x*_2_ → *r* engendered by the 2D coordinate transformation, and combining with the measured spatial chirp *x*_2_(*λ*) at its input, we obtain the radial chirp *k*_*r*_(*λ*) as shown in Fig. 4b (Supplementary Fig. 15). We find at each radial position a narrow spectrum (*δλ* ≈ 50 pm) whose central wavelength *λ*_c_ shifts quadratically with *r*, but with differently signed curvature for the superluminal and subluminal cases (Fig. 4c).

#### Propagation-invariance of the intensity distribution

The time-averaged intensity profile *I*(*x*, *y*, *z*) ∝ ∫*dt*∣*E*(*x*, *y*, *z*; *t*)∣^2^ is captured by scanning a CCD camera along the propagation axis *z* after the Fourier transforming lens (Fig. 3). For each wave packet, we plot in Fig. 5 the intensity distribution (at a fixed axial plane *z* = 30 mm) in transverse and meridional planes. As a point of reference, we start with a pulsed Bessel beam whose spatio-temporal spectrum is separable, where the spatial bandwidth is Δ*k*_*r*_ = 0.02 rad/*μ*m and is centered at *k*_*r*_ ≈ 0.06 rad/μm (Fig. 5a). Here, the full temporal bandwidth Δ*λ* is associated with each spatial frequency *k*_*r*_. The finite spatial bandwidth Δ*k*_*r*_ renders the propagation distance finite^56^, and we observe a Bessel beam comprising a main lobe of width Δ*r* ≈ 30 μm (FWHM) accompanied by several side lobes, which propagates for a distance ${L}_{\max }\,\approx\, 50$ mm. For comparison, the Rayleigh range of a Gaussian beam with a similar size and central wavelength is *z*_R_ ≈ 1 mm. By further increasing Δ*k*_*r*_ to 0.07 rad/*μ*m while remaining centered at *k*_*r*_ ≈ 0.06 rad/μm as shown in Fig. 5b, the axial propagation distance is reduced proportionately to ${L}_{\max }\,\approx\, 15$ mm, and the side lobes are diminished.

Now, rather than the separable spatio-temporal spectra for pulsed Bessel beams (Fig. 5a, b), we utilize the structured spatio-temporal spectra associated with 3D ST wave packets in which each *k*_*r*_ is associated with a single *λ* (Fig. 4), whose spatial bandwidths are all Δ*k*_*r*_ = 0.07 rad/*μ*m centered at *k*_*r*_ ≈ 0.06 rad/μm, similarly to the pulsed Bessel beam in Fig. 5b. Despite the large spatial bandwidth, the one-to-one correspondence between *k*_*r*_ and *λ* curtails the effect of diffraction, leading to an increase in the propagation distance (Fig. 5c–e). The subluminal 3D ST wave packet ($\widetilde{v}\,=\,0.83c$) in Fig. 5c propagates for ${L}_{\max }\,\approx\, 60$ mm, which is a 4 × improvement compared with the separable Bessel beam and a 60 × improvement compared with a Gaussian beam of the same spatial bandwidth. We observe a similar behavior for a superluminal 3D ST wave packet ($\widetilde{v}\,=\,1.37c$) in Fig. 5d, and a superluminal ST-OAM wave packet ($\widetilde{v}\,=\,1.16c$) with *ℓ* = 1 in Fig. 5e.

#### Reconstructing the spatio-temporal profile and measuring the group velocity

The spatio-temporal intensity profile *I*(*x*, *y*, *z*; *t*) = ∣*E*(*x*, *y*, *z*; *t*)∣^2^ of the 3D ST wave packet is reconstructed by placing the synthesizer (Fig. 3) in one arm of a Mach-Zehnder interferometer, while the initial 100-fs plane-wave pulses from the laser traverse an optical delay line *τ* in the reference arm (Fig. 6a). By scanning *τ* we reconstruct the spatio-temporal intensity profile in a meridional plane from the visibility of spatially-resolved interference fringes recorded by a CCD camera when the 3D ST wave packet and the reference pulse overlap in space and time. The reconstructed time-resolved intensity profile *I*(0, *y*, *z*; *t*) of the 3D ST wave packets corresponding to those in Fig. 5c–e are plotted in Fig. 6b–d at multiple axial planes, which reveal clearly the expected X-shaped profile that remains invariant over the propagation distance ${L}_{\max }$. In all cases, the on-axis pulse width, taken as the FWHM of *I*(0, 0, 0; *t*), is Δ*t* ≈ 6 ps. The spatio-temporal intensity profile of the superluminal ST-OAM wave packet with *ℓ* = 1 in Fig. 6d reveals a similar X-shaped profile, but with a central null instead of a peak, as expected from the helical phase structure associated with the OAM mode.

A subtle distinction emerges between the subluminal and superluminal wave packets regarding the axial evolution of their spatio-temporal profile. It can be shown that in presence of finite spectral uncertainty *δλ*, the realized ST wave packet can be separated into the product of an ideal ST wave packet traveling indefinitely at $\widetilde{v}$ and a long ‘pilot envelope’ traveling at *c*. The finite propagation distance ${L}_{\max }$ is then a consequence of temporal walk-off between the ST wave packet and the pilot envelope^52^. For subluminal ST wave packets, this results initially in a ‘clipping’ of the leading edge of the wave packet in (Fig. 6b at *z* = 20 mm), and ultimately a clipping of the trailing edge of the ST wave packet as the faster pilot envelope catches up with it (Fig. 6b at *z* = 40 mm). The opposite behavior occurs for the superluminal ST wave packet in Fig. 6c, d.

This experimental methodology also enables us to estimate the group velocity $\widetilde{v}$^28,30^. After displacing the CCD camera until the interference fringes are lost due to the mismatch between $\widetilde{v}\,=\,c\tan \theta$ for the ST wave packets and the reference pulses traveling at $\widetilde{v}\,=\,c$, we restore the interference by inserting a delay Δ*t* (Fig. 6e), which allows us to estimate $\widetilde{v}$ for the 3D ST wave packet. By tuning *B*, we record a broad span of group velocities in the range from $\widetilde{v}\ \approx \ 0.7c$ to $\widetilde{v}\ \approx \ 1.8c$ in free space (Fig. 6f). The continuous tunability of the group velocity of 3D ST wave packets over the subluminal and superluminal ranges allows them to be exploited in applications previously proposed for ST light-sheets, such as for constructing in-line optical delay lines for all-optical communications^29^, whereby the localization of 3D ST wave packets in both transverse dimensions can provide a significant advantage with regards to efficiently coupling into optical fibers.

#### Field amplitude and phase measurements

Lastly, we modify the measurement system in Fig. 6a by adding a small relative angle between the propagation directions of the 3D ST wave packets and the reference pulses, and make use of off-axis digital holography^57^ to reconstruct the amplitude ∣*ψ*(*x*, *y*, *z*; *τ*)∣ and phase *ϕ*(*x*, *y*, *z*; *τ*) of their complex field envelope *ψ*(*x*, *y*, *z*; *t*) = ∣*ψ*(*x*, *y*, *z*; *t*)∣*e*^*iϕ*(*x*, *y*, *z*; *t*)^ (Supplementary Note 3D). We reconstruct the complex field at a fixed axial plane *z* = 30 mm for the time delays: *τ* = −5, 0, and 5 ps (Fig. 7). First, we plot the results for ∣*ψ*(*x*, *y*, *z*; *τ*)∣ and phase *ϕ*(*x*, *y*, *z*; *τ*) for a superluminal 3D ST wave packet ($\widetilde{v}=1.1c$) with no OAM (*ℓ* = 0). At the pulse center *τ* = 0, the field is localized on the optical axis, whereas at *τ* = ±5 ps the field spreads away from the center (Fig. 7a)]. For *τ* ≠ 0 we find a spherical transverse phase distribution that is almost flat at *τ* = 0, similar to what one finds during the axial evolution of a Gaussian beam in space through its focal plane^14^.

After adding the OAM mode *ℓ* = 1 to the field structure, a similar overall behavior is observed for the superluminal ST-OAM wave packet except for two significant features. First, a dip is observed on-axis in Fig. 7b, in lieu of the central peak in Fig. 7a, as a result of the phase singularity associated with the OAM mode. Second, the phase at the wave-packet center *ϕ*(*x*, *y*, *z*; 0) at *z* = 30 mm is almost flat, while a helical phase front corresponding to OAM of order *ℓ* = 1 emerges as we move away from *τ* = 0. Finally, we plot in Fig. 7c, d iso-amplitude surface contours (0.6 × and 0.15 × the maximum amplitude ${I}_{\max }$) for the two 3D ST wave packets in Fig. 7a, b. We find a closed surface in Fig. 7c when *ℓ* = 0, and a doughnut structure in Fig. 7d when *ℓ* = 1 for the first contour $I\,=\,0.6{I}_{\max }$ that captures the structure of the wave-packet center. The second contour for $I\,=\,0.15{I}_{\max }$ captures the conic structure emanating from the wave-packet center that is responsible for the characteristic X-shaped spatio-temporal profile of all propagation-invariant wave packets in the paraxial regime.

## Discussion

We have demonstrated a general procedure for spatio-temporal spectral modulation of pulsed optical fields that is capable of synthesizing 3D ST wave packets localized in all dimensions. At the heart of our experimental methodology lies the ability to sculpt the angular dispersion of a generic optical pulse in two transverse dimensions. Crucially, this approach produces the non-differentiable angular-dispersion necessary for propagation invariance^21^. Because such a capability has proven elusive to date, AD-free X-waves have been the sole class of 3D propagation-invariant wave packets conclusively produced in free space. Unfortunately, X-waves can exhibit only minuscule changes in the group velocity with respect to *c* (typically ${{\Delta }}\widetilde{v}\, {\sim} \,0.001c$) in the paraxial regime, and only superluminal group velocities are supported. Furthermore, ultrashort pulses of width < 20 fs are required to observe a clear X-shaped profile^10^, and OAM-carrying X-waves have not been realized to date. Even more stringent requirements are necessary for producing focus-wave modes, and consequently they have not been synthesized in three dimensions to date. By realizing instead propagation-invariant 3D ST wave packets, an unprecedented tunable span of group velocities has been realized, clear X-shaped profiles are observed with pulse widths in the picosecond regime, and they outperformed spectrally separable pulsed Bessel beams of the same spatial bandwidth with respect to their propagation distance and transverse side-lobe structure. In addition, we demonstrated propagation-invariant ST-OAM wave packets with tunable group velocity in free space.

Further optimization of the experimental layout is possible. We made use of four phase patterns to produce the target spatio-temporal spectral structure. It is conceivable that this spectral modulation scheme can be performed with only three phase patterns, or perhaps even fewer. Excitingly, a new theoretical proposal suggests that a single non-local nanophotonic structure can produce 3D ST wave packets through a process of spatio-temporal spectral filtering^58^. This theoretical proposal indicates the role nanophotonics is poised to play in reducing the complexity of the synthesis system, potentially without recourse to filtering strategies.

Finally, efforts in the near future will be directed to reducing the spectral uncertainty *δλ* and concomitantly approaching *θ* → 45° to increase the propagation length to the kilometer range^24^. The experimental procedure presented here can in principle be extended to the synthesis of other exotic variants of ST wave packet, such as abruptly focusing needle pulses^59^ among other possibilities^19,60^. With access to 3D ST wave packets, previous work on guided ST modes in planar wave-guides^61^ can be extended to conventional single-mode and multi-mode waveguides^62^, and potentially to optical fibers^63–66^. Moreover, the localization in both transverse dimensions provided by 3D ST wave packets opens new avenues for nonlinear optics by increasing the intensity with respect to 2D ST wave packets, for introducing topological features such as spin texture in momentum space^58^, and for the exploration of spatio-temporal vortices and polarization singularities^67^. Our findings point therefore to profound new opportunities provided by the emerging field of space-time optics^58,61,62,68–71^.

## Methods

The 2D transformation used to construct the 3D STWP can be implemented by making use of *diffractive* optics^53,55,72,73^ or *refractive* optics^54^. We exploited both types of phase plates in our experiments to imprint the desired phase profiles: diamond-edged refractive phase plates^54^ and analog diffractive phase plates^55^.

### Refractive phase plates

The refractive optical elements used in our experiments are similar to those outlined by Lavery et al. in^54^, in which the transformation parameters are *C* = 4.77 mm, $D\,=\,\frac{3.2}{\pi }\approx \,1$ mm, and *d*_2_ = 310 mm. Each phase plate is made of the polymer PMMA (Poly methyl methacrylate) with accurately manufactured height profiles *Z*_1_(*x*_3_, *y*_3_) and *Z*_2_(*x*_4_, *y*_4_) to imprint the required phase profiles. The phase encountered by light at a wavelength *λ* traversing a height *Z* of a material of refractive index *n* – with respect to the phase encountered over the same distance in vacuum – is given by Φ = 2*π*(*n* − 1)*Z*/*λ*. Thus, the height profile of the first element is ${Z}_{1}({x}_{3},\ {y}_{3})=\frac{\lambda }{2\pi (n-1)}{{{\Phi }}}_{3}({x}_{3},\ {y}_{3})$ (Supplementary Fig. 14a) and that of the profile of the second element is ${Z}_{2}({x}_{4},\ {y}_{4})=\frac{\lambda }{2\pi (n-1)}{{{\Phi }}}_{4}({x}_{4},\ {y}_{4})$ (Supplementary Fig. 14b). Note that each surface height is wavelength-independent, and dispersion effects in the material manifest themselves as a change in the focal length *d*_2_ of the integrated lens for different wavelengths. Hence, in the experiment the system can be tuned to a specific wavelength by changing the distance between the two elements.

The elements were diamond-machined using a Natotech, 3-axis (X,Z,C) ultra precision lathe (UPL) in combination with a Nanotech NFTS6000 fast tool servo (FTS) system. The machined PMMA surfaces had a radius of 5.64 mm, angular spacing 1°, radial spacing of 5 μm, a spindle speed of 500 RPM, a roughing feed rate 5 mm/minute with a cut depth of 20 μm, and a finishing feed rate of 1 mm/minute with a cut depth of 10 μm^74^. The total sag height difference for each part was relatively small (≈115 μm for surface 1 and ≈144 μm for surface 2). The transmission efficiency of the combination of the elements is ≈85%.

#### Diffractive phase plates

The diffractive phase plates were fabricated in fused silica using Clemson University facilities. The fabrication process is outlined in^75^, which involves writing a binary phase grating on a stepper mask with an electron-beam and subsequently transferring this analog mask into a fused silica substrate with projection lithography. The phase grating period is designed to be larger than the cutoff period of the projection stepper for higher diffraction orders, so only the zeroth-order diffracted light from the stepper can be transmitted. The transmission coefficient of the stepper light is then a function of the duty cycle of the electron-beam-patterned binary phase grating. The spatial intensity distribution of light in the wafer plane can be controlled with a spatial duty cycle function, which then exposes the I-line resist with a spatially varying analog intensity profile. This allows fabrication of analog diffractive optics with a single exposure from the stepper rather than binary 2^*n*^ diffractive optics, resulting in high-efficiency optics. The transmission efficiency of the combination of the two faces is ≈ 92%.

The design parameters for the analog diffractive phase plates are chosen as follows: $D\,=\,\frac{7}{\pi }\,\approx \,2.2$ mm, *C* = 6 mm, *λ*_o_ = 798 nm, and *d*_2_ = 225 mm. These design parameters were optimized so the paraxial approximation remains valid over the desired transformation range of 5 mm.

## Supplementary information

Supplementary Information

## Data Availability

The data that support the plots within this paper and other findings of this study are available from the corresponding author upon reasonable request.

## Code availability

The software code used for data acquisition and data analysis are available from the corresponding author upon reasonable request.
