# Supplementary material for: Space-time wave packets localized in all dimensions
Source: Nat Commun. 2022 Aug 5;13:4573. doi: 10.1038/s41467-022-32240-0 (PMC9356057; doi:10.1038/s41467-022-32240-0)
Supplement: Supplementary file 1 — Supplementary Information [file 41467_2022_32240_MOESM1_ESM.pdf]

Supplementary Information:
Space-time wave packets localized in all dimensions

Murat Yessenov[1,†], Justin Free[2], Zhaozhong Chen[3], Eric G. Johnson[2], Martin P. J. Lavery[3], Miguel A. Alonso[4,5], and Ayman F. Abouraddy[1,*]

[1] *CREOL, The College of Optics & Photonics, University of Central Florida, Orlando, Florida 32816, USA*

[2] *Micro-Photonics Laboratory, the Holcombe Department of Electrical and Computer Engineering, Clemson University, Clemson, South Carolina 29634, USA*

[3] *James Watt School of Engineering, University of Glasgow, UK*

[4] *CNRS, Centrale Marseille, Institut Fresnel, Aix Marseille Univ., Marseille, France*

[5] *The Institute of Optics, University of Rochester, Rochester, NY, USA*

(Dated: July 22, 2022)

* Corresponding authors: raddy@creol.ucf.edu; †yessenov@knights.ucf.edu

CONTENTS

Supplementary Note 1. Representation of 3D space-time wave packets on the surface of the light-cone 3
A. Light-cone representation for 2D ST wave packets (light sheets) 3
B. Spectral representation for 3D fields on the light-cone surface 6
1. Conventional optical fields 6
2. 3D ST wave packets 11

Supplementary Note 2. Synthesis of 3D space-time wave packets 15
A. Spectral analysis: Volume chirped Bragg grating (CBG) 15
1. Introducing spatial chirp 15
2. Spectral uncertainty induced by CBGs 19
B. Tunable 1D spectral transformation 20
C. Fixed 2D coordinate transformation 22
D. Spatial Fourier transform 26
E. Full system analysis 26
F. Synthesizing pulsed Bessel beams with separable spatio-temporal spectrum 27

Supplementary Note 3. Characterization of space-time wave packets 28
A. Spatio-temporal spectrum measurements 28
B. Time-averaged intensity measurements 29
C. Time-resolved intensity measurements 29
D. Measurements of the field amplitude and phase for 3D ST wave packets 31

References 34

Supplementary References 34

Supplementary Note 1. REPRESENTATION OF 3D SPACE-TIME WAVE PACKETS ON THE SURFACE OF THE LIGHT-CONE

In our previous work on space-time (ST) wave packets in the form of light-sheets [1–6], we make heavy use of the representation of the wave-packet spectral support domain on the surface of the light-cone. This is a useful visualization tool that provides physical intuition with regards to the structure and behavior of ST wave packets. In this Section, we briefly review this representation in the reduced-dimension case of ST light sheets [2], where the field is localized along one transverse dimension and extended uniformly along the other. We refer to these field structures as 2D ST wave packets (one transverse dimension and one longitudinal dimension). We then proceed to show that such a representation can also be gainfully employed with minor changes for ST wave packets localized in all dimensions. We refer to such field structures as 3D ST wave packets (two transverse dimensions and one longitudinal dimensions). Therefore, the wealth of results that have amassed over the past few years based on this conceptual framework [6] can be appropriated for the new 3D ST wave packets investigated here.

A. Light-cone representation for 2D ST wave packets (light sheets)

When the field is held uniform along one transverse dimension (say y), then the dispersion relationship in free space is $k_x^2+k_z^2=(\frac{\omega}{c})^2$, where k_x and k_z are the transverse and longitudinal components of the wave vector along x and z, respectively, ω is the temporal frequency, and c is the speed of light in vacuum. This relationship is represented geometrically by the surface of a cone that we refer to as the light-cone. A monochromatic plane wave $e^{i(k_xx+k_zz-\omega t)}$ is represented by a point on the surface of the light-cone (Supplementary Fig. 1). In general, a pulsed beam $E(x,z;t)$ is expressed as a product of a slowly varying envelope $\psi(x,z;t)$ and a carrier term $e^{i(k_o z-\omega_o t)}$, where ω_o is a fixed temporal frequency, and $k_o=\omega_o/c$ is its associated wave number. The envelope is written in terms of an angular spectrum as follows:

$$\psi(x,z;t)=\iint dk_x d\Omega \widetilde{\psi}(k_x,\Omega)e^{i\{k_xx+(k_z-k_o)z-\Omega t\}}, \tag{S1}$$

where $\Omega=\omega-\omega_o$, and the spatio-temporal spectrum $\widetilde{\psi}(k_x,\Omega)$ is the 2D Fourier transform of $\psi(x,0;t)$. The spectral support domain for a pulsed beam or wave packet corresponds in general to a 2D area on the surface of the light-cone [2]; see Supplementary Fig. 3.

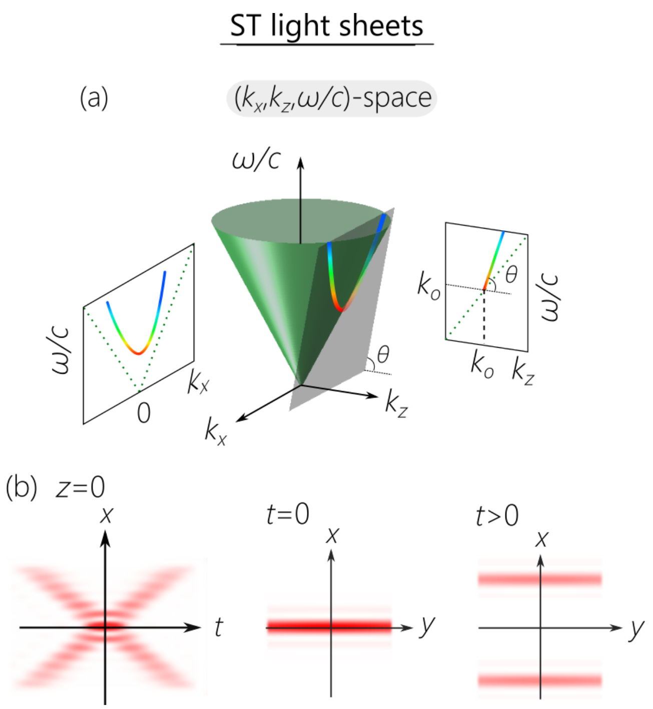

Supplementary Fig. 1. Representation of the spectral support domain for 2D ST wave packets (light sheets) on the light-cone surface. (a) The spatio-temporal spectrum is restricted to the intersection of the light-cone $k_x^2+k_z^2=(\frac{\omega}{c})^2$ with a tilted spectral plane in the region $k_z>0$. The spectral projection onto the $(k_z,\frac{\omega}{c})$-plane is a straight line, and that onto the $(k_x,\frac{\omega}{c})$-plane is a segment of a conic section. (b) The spatio-temporal intensity profile in the plane $z=0$, $I(x,z=0;t)$, and the transverse intensity profile in the same $z=0$ plane at the wave-packet center $t=0$, and off-center $t>0$. In the (x,t)-plane, the spatio-temporal intensity profile is X-shaped. In the (x,y)-plane, the 2D ST wave packet at $t=0$ takes the form of a light sheet that is localized along the x-axis and extends uniformly along the y-axis.

For the special case of a propagation-invariant 2D ST wave packet, the spectral support domain is confined to the intersection of the light-cone with a spectral plane that is parallel to the k_x-axis and makes an angle θ with the k_z-axis, which is thus given by the equation:

$$\Omega=(k_z-k_o)c\tan\theta, \tag{S2}$$

where we refer to θ as the spectral tilt angle. Consequently, there is a one-to-one relationship between Ω and $|k_x|$, which takes the form of a parabola in the narrowband ($\Delta\omega\ll\omega_o$, where $\Delta\omega$ is the temporal bandwidth) and paraxial ($\Delta k_x\ll k_o$, where Δk_x is the spatial bandwidth) limits:

$$\frac{\Omega}{\omega_o}=\frac{k_x^2}{2k_o^2(1-\widetilde{n})}, \tag{S3}$$

where $\widetilde{n}=\cot\theta$ is the wave packet group index in free space. Because of this correspondence between Ω and $|k_x|$, the spatio-temporal spectrum has a reduced dimensionality with respect to a conventional pulsed beam $\widetilde{\psi}(k_x,\Omega)\rightarrow\widetilde{\psi}(k_x)\delta(\Omega-\Omega(k_x))$, where $\Omega=\Omega(k_x)$ is given by Eq. S3. The wave-packet envelope now takes the simpler integral form:

$$\psi(x,z;t)=\int dk_x\,\widetilde{\psi}(k_x)\,e^{ik_xx}\,e^{-i(t-z/\widetilde{v})\Omega(k_x)}=\psi(x,0;t-z/\widetilde{v}), \tag{S4}$$

which is a 2D ST wave packet that travels rigidly in free space at a group velocity $\widetilde{v}=c\tan\theta$.

The spectral projection of the 2D ST wave packet onto the $(k_z,\frac{\omega}{c})$-plane is a straight line that makes an angle θ with the k_z-axis and intersects with the light-line $k_z=\frac{\omega}{c}$ at the point $(k_z,\frac{\omega}{c})=(k_o,k_o)$. The corresponding spectral projection onto the $(k_x,\frac{\omega}{c})$-plane is a segment of a conic section: an ellipse when $0^\circ<\theta<45^\circ$ or $135^\circ<\theta<180^\circ$, whereupon $|\widetilde{v}|<c$; a hyperbola when $45^\circ<\theta<135^\circ$, whereupon $|\widetilde{v}|>c$; a straight tangent line when $\theta=45^\circ$ and $\widetilde{v}=c$, corresponding to a plane-wave pulse; and a parabola when $\theta=135^\circ$ and $\widetilde{v}=-c$. We refer to ST wave packets associated with the range $0^\circ<\theta<45^\circ$ as subluminal, with $45^\circ<\theta<90^\circ$ as superluminal, and with $\theta>90^\circ$ (whereupon $\widetilde{v}<0$) as negative-$\widetilde{v}$ ST wave packets.

Note that causal emission and propagation require that only the values $k_z>0$ be considered, so that the light-cone half corresponding to the acausal backward-propagating components $k_z<0$ is eliminated from consideration [4, 7].

The one-to-one correspondence between $|k_x|$ and ω has a crucial impact on the form of the axial evolution of the time-averaged intensity $I(x,z)=\int dt|E(x,z;t)|^2=\int dt|\psi(x,z;t)|^2$. Substituting for $\psi(x,z;t)$ from Eq. S4 we reach:

$$I(x,z)=2\pi k_o^2(1-\tilde{n})\left[\int dk_x|\widetilde{\psi}(k_x)|^2+\int dk_x\widetilde{\psi}(k_x)\widetilde{\psi}^*(-k_x)e^{i2k_xx}\right]=I_o+I(2x), \tag{S5}$$

where $I_o=2\pi k_o^2(1-\tilde{n})\int dk_x|\widetilde{\psi}(k_x)|^2$ and $I(x)$ is the Fourier transform of $\widetilde{\psi}(k_x)\widetilde{\psi}^*(-k_x)$. In other words, $I(x,z)$ is altogether independent of the axial coordinate z, and is formed of the sum of a constant background pedestal term I_o and a localized spatial feature at $x=0$. Moreover, the height of the localized spatial feature cannot exceed the height of the pedestal. This structure of

the intensity profile for 2D ST wave packets has been borne out in previous measurements [2, 5]. It is important to note that this structure is unique to 2D ST wave packets. The time-averaged intensity of 3D ST wave packets cannot be separated into a sum of a pedestal and a localized central feature (see main text).

B. Spectral representation for 3D fields on the light-cone surface

1. *Conventional optical fields*

When both transverse coordinates x and y are retained, we have the general dispersion relationship $k_x^2+k_y^2+k_z^2=(\frac{\omega}{c})^2$ in free space. This relationship is represented mathematically by a hypercone in 4D, which cannot be visualized in 3D space. However, because we are mostly interested in cylindrically symmetric fields, we can write the dispersion relationship as $k_r^2+k_z^2=(\frac{\omega}{c})^2$, where $k_r=\sqrt{k_x^2+k_y^2}$ is the radial wave number. This dispersion relationship can indeed be represented in 3D space. Because k_r is positive-valued only, in contrast to k_x in the case of the 2D ST wave packets that can take on either positive or negative values, only the quarter of the light-cone corresponding to $k_r>0$ *and* $k_z>0$ need be retained here.

A wave packet is once again given in terms of a carrier term and a slowly varying envelope $E(x,y,z;t)=Re\left[e^{i(k_{o}z-\omega_{o}t)}\psi(x,y,z;t)\right]$, where:

$$\psi(x,y,z;t)=\iiint dk_x dk_y d\Omega\,\widetilde{\psi}(k_x,k_y,\Omega)\,e^{i\{k_x x+k_y y+(k_z-k_{o})z-\Omega t\}}, \tag{S6}$$

and $\widetilde{\psi}(k_x,k_y,\Omega)$ is the 3D Fourier transform of $\psi(x,y,0;t)$. We switch to transverse polar coordinates (r,φ) in physical space and to the corresponding polar coordinates (k_r,χ) in Fourier space. In physical space we have the relationships:

$$r=\sqrt{x^2+y^2},\ \varphi=\arctan\left(\frac{y}{x}\right),\ x=r\sin\varphi,\ y=r\cos\varphi; \tag{S7}$$

and in Fourier space we have

$$k_r=\sqrt{k_x^2+k_y^2},\ \chi=\arctan\left(\frac{k_y}{k_x}\right),\ k_x=k_r\sin\chi,\ k_y=k_r\cos\chi. \tag{S8}$$

We can thus rewrite the angular spectrum of the envelope as follows:

$$\psi(r,\varphi,z;t)=\iiint dk_r d\chi d\Omega\,k_r\widetilde{\psi}(k_r,\chi,\Omega)\,e^{ik_r r\cos(\varphi-\chi)}\,e^{i(k_z-k_{o})z}e^{-i\Omega t}; \tag{S9}$$

where the integral over χ extends from 0 to 2π, that over k_r extends from 0 to ∞, and that for Ω from $-\Delta\omega/2$ to $\Delta\omega/2$.

We can separate the spatio-temporal spectrum $\widetilde{\psi}(k_r,\chi,\Omega)$ with respect to the radial and azimuthal coordinates k_r and ξ, respectively, as follows:

$$\widetilde{\psi}(k_r,\chi,\Omega)=\frac{1}{2\pi}\sum_{\ell=-\infty}^{\infty}\widetilde{\psi}_{\ell}(k_r,\Omega)e^{i\ell\chi}, \tag{S10}$$

whereupon the wave packet envelope can be expressed as:

$$\psi(r,\varphi,z;t)=\sum_{\ell=-\infty}^{\infty}e^{i\ell\varphi}\iint dk_r d\Omega\, k_r\widetilde{\psi}(k_r,\Omega)J_{\ell}(k_r r)e^{i(k_z-k_o)z}e^{-i\Omega t}; \tag{S11}$$

where we have made use of the identity $2\pi J_{\ell}(x)=\int_{-\pi}^{\pi}dy e^{i(x\sin y-\ell y)}$, and $J_{\ell}(\cdot)$ is the ℓth-order Bessel function of the first kind.

One simplification is obtained by assuming that the spatio-temporal spectrum is azimuthally symmetric, which corresponds to setting $\ell=0$, and the summation over ℓ is removed. This assumption is equivalent to setting $\widetilde{\psi}(k_r,\chi,\Omega)\rightarrow\widetilde{\psi}(k_r,\Omega)$, which is thus independent of χ, whereupon:

$$\psi(r,\varphi,z;t)=\iint dk_r d\Omega\, k_r\widetilde{\psi}(k_r,\Omega)J_0(k_r r)\, e^{i(k_z-k_o)z}e^{-i\Omega t}=\psi(r,z;t), \tag{S12}$$

where $J_0(\cdot)$ is the zeroth-order Bessel function of the first kind. As a result of the azimuthal symmetry of the spectrum, the wave-packet envelope is also azimuthally symmetric in physical space. We will address shortly the more general case of fields with azimuthal variation.

Comparing Eq. S1 for $\psi(x,z;t)$ to Eq. S12 for $\psi(r,z;t)$, we find that both have 2D spatio-temporal spectra: $\widetilde{\psi}(k_x,\Omega)$ for the former and $\widetilde{\psi}(k_r,\Omega)$ for the latter. The light-cone in $(k_r,k_z,\frac{\omega}{c})$-space can thus be used to represent the spectral support domain for $\psi(r,z;t)$. Here, each point on the light-cone at coordinates $(k_r,k_z,\frac{\omega}{c})$ corresponds to a monochromatic Bessel beam $E(r,z;t)=J_0(k_r r)e^{i(k_z z-\omega t)}$ rather than a monochromatic plane wave; see Supplementary Fig. 2.

In the case of light sheets in which the field is uniform along y, the light-cone in Supplementary Fig. 1(a) suffices to capture the complete description of the spectral support domain. On the other hand, in the case of fields in 3D space, the light-cone in Supplementary Fig. 2(a) does *not* convey the whole picture because we collapsed all the plane waves having spatial-frequency pairs (k_x,k_y) into their radial counterpart k_r. The picture can be completed by adding a second spectral representation in $(k_x,k_y,\frac{\omega}{c})$-space. Each point with coordinates $(k_x,k_z,\frac{\omega}{c})$ corresponds to a monochromatic plane wave $e^{i(k_x x+k_y y+k_z z-\omega t)}$, where $k_z=\sqrt{(\frac{\omega}{c})^2-k_x^2-k_y^2}$. Although k_z is not

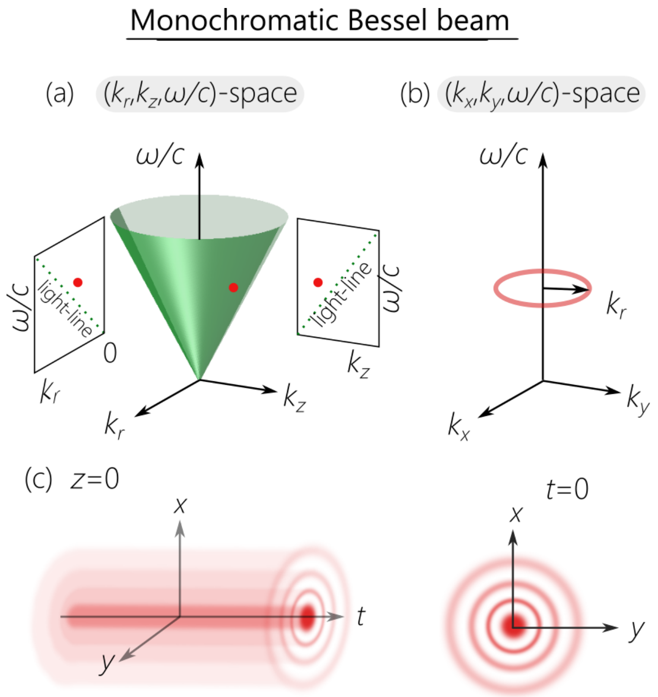

Supplementary Fig. 2. Representation of the spectral support domain of a monochromatic Bessel beam in 3D space on the surface of the light-cone. (a) A point on the surface of the light-cone $k_r^2+k_z^2=(\frac{\omega}{c})^2$ at coordinates $(k_r,k_z,\frac{\omega}{c})$ corresponds to a monochromatic Bessel beam of the form $E(r,z;t)=J_0(k_r r)e^{i(k_z z-\omega t)}$. (b) In $(k_x,k_y,\frac{\omega}{c})$-space, the point on the light-cone in (a) takes the form of a horizontal iso-frequency circle of radius k_r at a height $\frac{\omega}{c}$. (c) In physical space at an axial plane $z=0$, the intensity $I(x,y,0;t)$ is uniform along t and takes the form of a Bessel function $J_0^2(k_r r)$ in the (x,y)-plane at any t.

represented in this space explicitly, it can nevertheless be found by referring to the light-cone in Supplementary Fig. 2(a). The single point representing a monochromatic Bessel beam on the light-cone in Supplementary Fig. 2(a) corresponds to a horizontal circle of radius k_r in $(k_x,k_y,\frac{\omega}{c})$-space in Supplementary Fig. 2(b).

The representation in $(k_x,k_y,\frac{\omega}{c})$-space is particularly useful for wave packets in 3D space because it provides the spatio-temporal spectral structure required to synthesize the field in question. For a monochromatic Bessel beam, Supplementary Fig. 2(b) points to the well-known approach for producing such a beam by inserting a spatial filter in the Fourier domain in the form of a thin annulus, followed by a spherical converging lens [8].

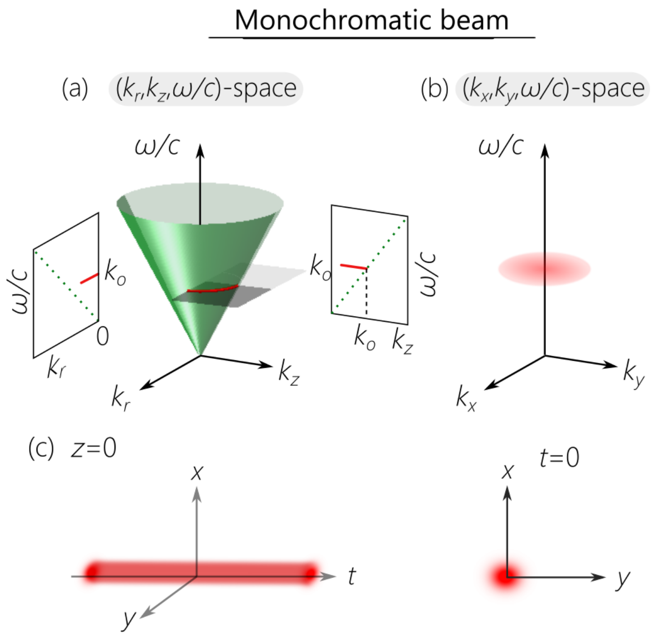

Supplementary Fig. 3. Representation of the spectral support domain for a monochromatic beam in 3D space on the surface of the light-cone. (a) The spectral support domain is restricted to the circle on the light-cone surface at its intersection with a horizontal iso-frequency plane $\omega{=}\omega_{\text{o}}$. (b) In $(k_x,k_y,\frac{\omega}{c})$-space, the spectral support domain is a horizontal iso-frequency disc at $\omega{=}\omega_{\text{o}}$. The disc radius corresponds to the maximum spatial frequency in (a). (c) In physical space at $z{=}0$, the intensity $I(x,y,0;t)$ is uniform along t with the transverse beam profile given by the plot in the (x,y)-plane at any t.

More generally, the spectral support domain for a monochromatic beam at frequency ω_{o} is restricted to the circle at the intersection of the light-cone with a horizontal iso-frequency plane $\omega{=}\omega_{\text{o}}$; see Supplementary Fig. 3(a). In $(k_x,k_y,\frac{\omega}{c})$-space, the spectral support domain lies in the horizontal plane $\omega{=}\omega_{\text{o}}$, and takes the form of a disc. The central point at $k_x{=}k_y{=}0$ corresponds to the point on the light-line in Supplementary Fig. 3(a). The radius of the disc in Supplementary Fig. 3(b) corresponds to the maximum radial spatial frequency in Supplementary Fig. 3(a).

As another example, consider a pulsed plane wave that lacks transverse spatial features. The spectral support domain of this field in $(k_r,k_z,\frac{\omega}{c})$-space lies along the light-line $k_z{=}\frac{\omega}{c}$ [Supplementary Fig. 4(a)], whereupon $\widetilde{\psi}(k_r,\Omega){\to}\widetilde{\psi}(\Omega)\frac{\delta(k_r)}{k_r}$, so that the pulse is a plane wave with no

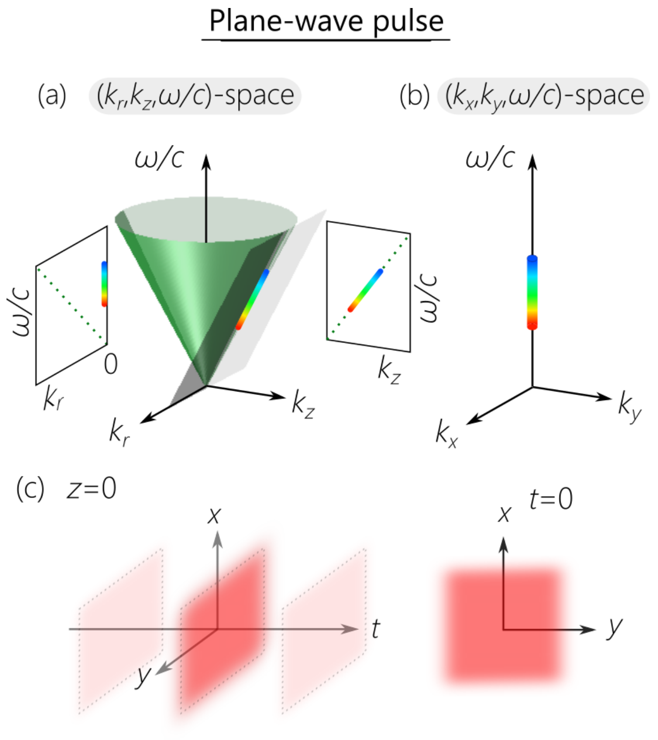

Supplementary Fig. 4. Representation of the spectral support domain for a plane-wave pulse in 3D space on the surface of the light-cone. (a) The spectral support domain is restricted to the straight-line tangent at k_r=0 on the light-cone surface. (b) In $(k_x,k_y,\frac{\omega}{c})$-space, the spectral support domain is a vertical line along the $\frac{\omega}{c}$-axis. (c) In physical space at an axial plane z=0, the intensity $I(x,y,0;t)$ is uniform everywhere at fixed t. The overall intensity drops away from t=0 according to the pulse linewidth.

transverse features, and whose longitudinal and temporal shape is determined exclusively by $\widetilde{\psi}(\Omega)$, therefore:

$$\psi(z;t)=\int d\Omega\widetilde{\psi}(\Omega)e^{i\Omega(t-z/c)}=\psi(0;t-z/c). \tag{S13}$$

The spectral support domain in $(k_x,k_y,\frac{\omega}{c})$-space lies along the $\frac{\omega}{c}$-axis, with k_x=k_y=0 [Supplementary Fig. 4(b)].

Finally, the most general conventional azimuthally symmetric pulsed beam or wave packet has a 2D spectral support domain on the surface of the light-cone in $(k_r,k_z,\frac{\omega}{c})$-space as shown in Supplementary Fig. 5(a). Typically, $\widetilde{\psi}(k_r,\Omega)$ is separable with respect to k_r and Ω, $\widetilde{\psi}(k_r,\Omega)\rightarrow\widetilde{\psi}_r(k_r)\widetilde{\psi}_t(\Omega)$. The spectral support domain in $(k_x,k_y,\frac{\omega}{c})$-space as shown in Supplementary Fig. 5(b)

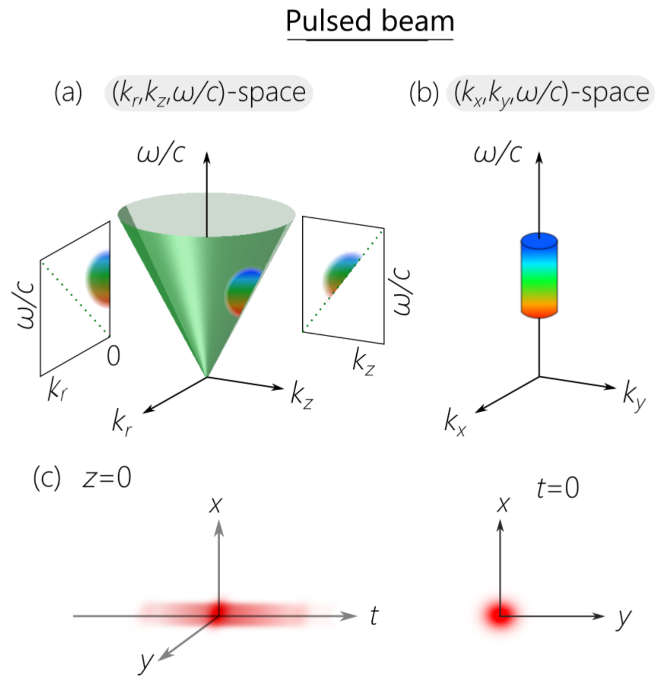

Supplementary Fig. 5. Representation of the spectral support domain for a conventional pulsed beam in 3D space on the surface of the light-cone. (a) The spectral support domain is a 2D area on the light-cone surface. (b) In $(k_x,k_y,\frac{\omega}{c})$-space, the spectral support domain is a cylindrical volume whose radius and height correspond to the spatial and temporal bandwidths of the wave packet. (c) In physical space at $z{=}0$, the intensity $I(x,y,0;t)$ varies in intensity along t according to the pulse linewidth, with the transverse beam profile as prescribed in the plot in the (x,y)-plane at $t{=}0$.

takes the form approximately of a cylindrical volume centered on the $\frac{\omega}{c}$-axis, whose radius is the maximum width of spatial spectrum $\widetilde{\psi}_r(k_r)$, and whose heights is the extent of the temporal spectrum $\widetilde{\psi}_t(\Omega)$.

2. *3D ST wave packets*

When considering 3D ST wave packets that are localized along all dimensions, such that the time-averaged intensity takes the form of an axial needle [9–11], the spectral support domain is once again restricted to the intersection of the light-cone $k_r^2{+}k_z^2{=}(\frac{\omega}{c})^2$ with the spectral plane $\Omega{=}(k_z{-}k_o)c\tan\theta$, as in the case of the 2D ST wave packets, where the plane is parallel to the

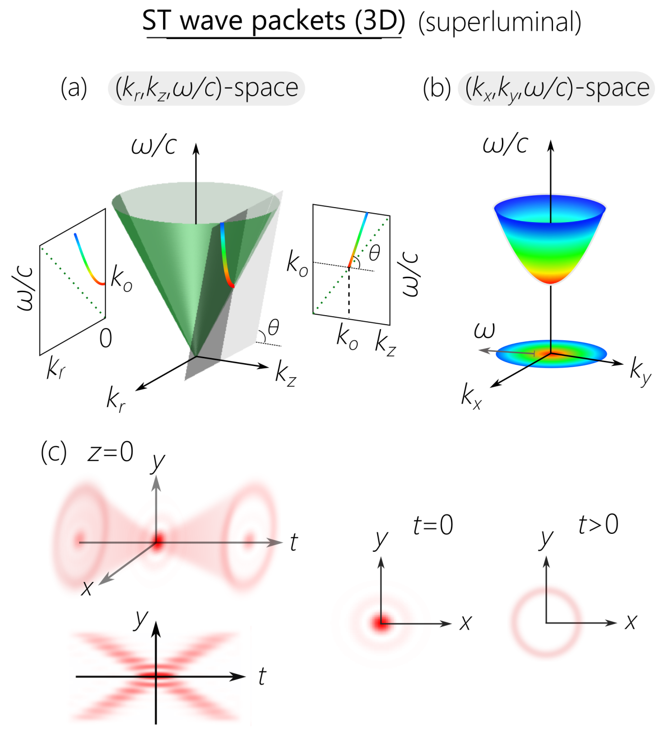

Supplementary Fig. 6. Representation of the spectral support domain for a superluminal 3D ST wave packet on the surface of the light-cone. (a) The spectral support domain is restricted to the hyperbola on the light-cone surface at its intersection with a spectral plane that is parallel to the k_r-axis and makes an angle θ with the k_z-axis ($45^\circ<\theta<90^\circ$). (b) In $(k_x,k_y,\frac{\omega}{c})$-space, the spectral support domain is a 2D surface in the form of one half of a two-sheet hyperboloid (an elliptic hyperboloid). (c) In physical space at $z=0$, the intensity $I(x,y,0;t)$ takes the form of two cones emanating from the central feature at $t=0$. In any meridional plane such as $x=0$, the spatio-temporal intensity profile is X-shaped. In the (x,y)-plane at $t=0$, the intensity profile is centered at $x=y=0$. At $t\neq0$, the profile in the (x,y)-plane is an annulus whose radius increases with t.

k_r-axis and makes an angle θ with the k_z-axis. The group velocity of the propagation-invariant 3D ST wave packet is $\widetilde{v}=c\tan\theta$:

$$\psi(r,\varphi,z;t)=\sum_{\ell=-\infty}^{\infty}e^{i\ell\varphi}\int dk_r k_r\widetilde{\psi}(k_r)J_\ell(k_r r)e^{-i\Omega(t-z/\widetilde{v})}=\psi(r,\varphi,0;t-z/\widetilde{v}). \quad \text{(S14)}$$

The impact of θ on the shape of the conic section resulting from this intersection is the same as that for the 2D ST wave packet or light sheets. We depict the superluminal scenario in Supplementary

Fig. 6(a) where $45^\circ<\theta<90^\circ$, $\widetilde{v}>c$, and the spectral support domain is a hyperbola. Alternatively, a subluminal wave packet where $0^\circ<\theta<45^\circ$ and $\widetilde{v}<c$ is shown in Supplementary Fig. 7(a), which has an ellipse for its spectral support domain. In the narrowband paraxial regime, the conic section can be approximated in the vicinity of $k_r=0$ by a parabola:

$$\frac{\Omega}{\omega_\mathrm{o}}=\frac{k_r^2}{2k_\mathrm{o}^2(1-\widetilde{n})}. \tag{S15}$$

Introducing this quadratic relationship between Ω and k_r into a pulsed beam is the goal of our synthesis methodology. Note that for small bandwidths, this also entails a quadratic relationship between $\lambda-\lambda_\mathrm{o}$ and k_r, where $\lambda_\mathrm{o}=\frac{2\pi}{k_\mathrm{o}}$.

In the case of the 3D ST wave packets, the structure of spectral representation in $(k_x,k_y,\frac{\omega}{c})$-space is particularly instructive, as shown in Supplementary Fig. 6(b) for the superluminal case and in Supplementary Fig. 7(b) for its subluminal counterpart. Consider first the superluminal 3D ST wave packet in Supplementary Fig. 6(b). Each point $(k_r,k_z,\frac{\omega}{c})$ along the hyperbola on the surface of the light-cone in Supplementary Fig. 6(a) corresponds to a circle of radius k_r at a height $\frac{\omega}{c}$ in Supplementary Fig. 6(b). Because each k_r is associated with a different ω, the circles in Supplementary Fig. 6(b) are all located at different heights, thus forming a 2D surface rather than the 3D volume in Supplementary Fig. 5(b) for a conventional pulsed beam. Because the spectral trajectory on the light-cone surface in Supplementary Fig. 6(a) is a hyperbola, the surface in Supplementary Fig. 6(b) is one half of a two-sheet hyperboloid (an elliptic hyperboloid) centered on the $\frac{\omega}{c}$-axis.

In physical space, as shown in Supplementary Fig. 6(c), the spatio-temporal intensity profile at a fixed axial profile $z=0$ takes the form of a central peak at $x=y=0$ and $t=0$, with two cones centered on the t-axis emanating from this peak. Consequently, in any meridional plane, for example $x=0$, the intensity profile is X-shaped, similarly to its 2D ST wave packet counterpart [Supplementary Fig. 1(c)]. The shape of the cross section of the intensity profile is time-dependent: at $t=0$ it is localized at $x=y=0$, at $t\neq0$ it takes the form of an annulus whose radius increases with t.

The corresponding graphs for a subluminal 3D ST wave packet are plotted in Supplementary Fig. 7(b,c). Because the intersection of the spectral plane with the light-cone is an ellipse, the spectral support domain in $(k_x,k_y,\frac{\omega}{c})$-space is an ellipsoid of revolution, or spheroid, as shown in Supplementary Fig. 7(b). Depending on the value of θ, this spheroid may be oblate of prolate. The threshold value of $\tan\theta=\frac{1}{\sqrt{2}}$ separates these two regimes. Indeed, at $\tan\theta=\frac{1}{\sqrt{2}}$, the projection

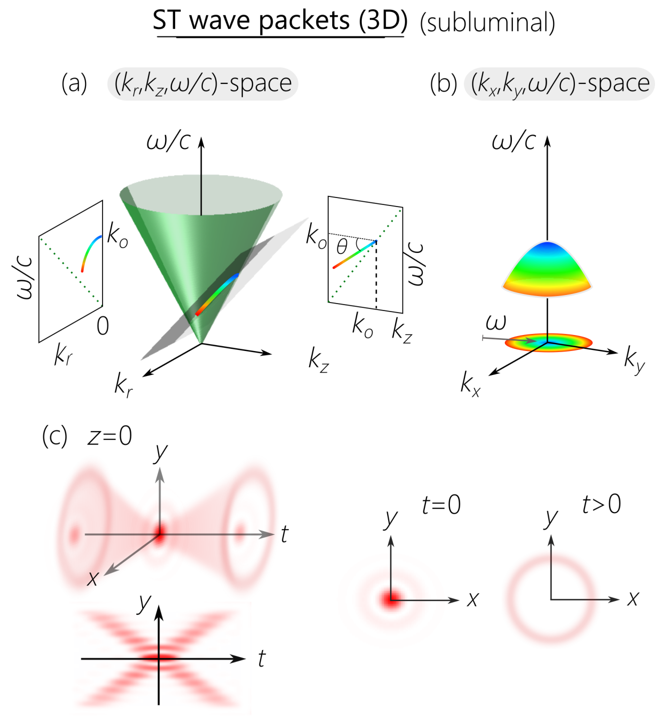

Supplementary Fig. 7. Representation of the spectral support domain for a subluminal 3D ST wave packet on the surface of the light-cone. (a) The spectral support domain is restricted to the ellipse on the light-cone surface at its intersection with a spectral plane that is parallel to the k_r-axis and makes an angle θ with the k_z-axis ($0°<\theta<45°$). (b) In $(k_x,k_y,\frac{\omega}{c})$-space, the spectral support domain is a 2D surface in the form of a ellipsoid of revolution (a spheroid). (c) In physical space at z=0, the intensity $I(x,y,0;t)$ takes the form of two cones emanating from the central feature at t=0. The structure of the spatio-temporal intensity profile is similar to that in Supplementary Fig. 6(c).

of the spectral support domain on the light-cone surface onto the $(k_r,\frac{\omega}{c})$-plane is a circle, and the spectral support domain in $(k_x,k_y,\frac{\omega}{c})$-space is a sphere. Of course, in all cases only the plane-wave components corresponding to $k_z>0$ are physically meaningful.

Lastly, the spatio-temporal intensity profile of a subluminal 3D ST wave packet [Supplementary Fig. 7(c)] in general resembles that of its superluminal counterpart [Supplementary Fig. 6(c)].

The dynamics of 3D ST wave packets upon free space propagation are calculated by applying Fresnel propagation to the wave packets after introducing the space-time coupling given in Eq. S15 into the optical field.

Supplementary Note 2. SYNTHESIS OF 3D SPACE-TIME WAVE PACKETS

The experimental methodology to synthesize 3D ST wave packets shown in Fig. 3 in the main text is expanded in more technical detail here in Supplementary Fig. 8. Our strategy consists of three stages as outlined in Supplementary Fig. 8(a):

1. Spectral analysis
2. A tunable 1D spectral transformation
3. A fixed 2D coordinate transformation

We start off with femtosecond plane-wave pulses from a mode-locked Ti:sapphire laser (Tsunami; Spectra Physics) of width $\approx$100 fs and bandwidth $\Delta\lambda\approx$10 nm centered at a wavelength of $\approx$800 nm. The pulses are directed to the first stage of the synthesis system: spectral analysis using a volume chirped Bragg grating (CBG), which spatially resolves the spectrum [12, 13]. A double-pass through the CBG produces a linear spatial chirp but with a flat phase front. The spectrally resolved wave front from the CBG arrangement is then fed to the second stage: a 1D conformal mapping implemented by a pair of spatial light modulators (SLM; Meadowlark 1920$\times$1080 series) that produces a logarithmic coordinate transformation to yield a logarithmic spatial chirp. The transformed wave front is then directed to a fixed log-polar-to-Cartesian coordinate transformation. This 2D coordinate transformation maps a line at its input into a circle at its output [14–16], and is implemented by means of two refractive [17] or diffractive [18] phase plates. The combination of the 1D spectral transformation and the 2D coordinate transformation produces a field endowed with a quadratic radial chirp. Finally, a spherical converging lens performs an optical Fourier transform along both transverse dimensions to yield 3D ST wave packets. We proceed to provide a detailed description of each stage of this novel spatio-temporal synthesis setup.

A. Spectral analysis: Volume chirped Bragg grating (CBG)

1. *Introducing spatial chirp*

The goal of the first stage in the setup is to spatially resolve the spectrum but retain a flat phase-front, which is necessary for the successful operation of the subsequent coordinate transformations.

A conventional surface grating is therefore *not* suitable for our purposes because the resolved spectrum does *not* have a flat phase. Instead, we make use of an arrangement based on a volume CBG to achieve this goal.

The CBG is a reflective Bragg grating [19] with a multilayered structure having a linearly varying periodicity $\Lambda(z)$ along the longitudinal axis z. Consequently, different wavelengths are reflected from different depths z within the grating volume [13]. As a result, the CBG introduces a *spectral chirp* into normally incident pulses, thus stretching the plane-wave pulse in time [Supplementary Fig. 9(a)]. For this reason, volume CBGs are widely used in high-power chirped pulse amplification (CPA) systems, where they are well-known for their high damage threshold and their ability to introduce extremely large spectral chirp [20, 21].

Our goal is to produce *spatial* chirp rather than *spectral* chirp. At oblique incidence, however, both spectral *and* spatial chirps are introduced; i.e., the pulse is stretched longitudinally in time, and the spectrum is resolved transversely in space [Supplementary Fig. 9(b)]. The spatial chirp can be determined easily from two parameters: (1) the chirp rate of the CBG structure $\beta = \frac{d\lambda}{dz} = \frac{1}{2n}\frac{d\Lambda}{dz}$, where n is the average refractive index of the CBG; and (2) the incident angle ϕ_1 with respect to the normal to the grating structure. After reflecting from the CBG at oblique incidence, the spectrum is spatially resolved along the x-axis such that each wavelength λ is located at a position $x(\lambda)$ given by:

$$x(\lambda) = \frac{1}{\beta}(\lambda - \lambda_o)\zeta(\phi_1), \tag{S16}$$

where $\zeta(\phi_1) = \frac{n \sin 2\phi_1}{n^2 - \sin^2\phi_1}$, $\lambda_o = 2n\Lambda_o$ is the central wavelength, $\Lambda_o = \Lambda(L/2)$ is the central periodicity of the CBG, and L is its length along the direction of the chirp. Here $x(\lambda)$ is the transverse spatial displacement each wavelength experiences with respect to λ_o.

For our purposes here, we aim at retaining the spatial chirp while eliminating the accompanying spectral chirp [12], which we achieve by directing the field to an identical CBG placed in a reversed geometry with respect to the first one [13]; see Supplementary Fig. 9(c). The output from CBG_1 first passes through a $4f$ imaging system that flips the field along x and thus reverses the sign of the spatial chirp. Consequently, after passing through CBG_2 with an opposite sign of chirp $\beta_2 = -\beta_1$, CBG_2 doubles the the spatial chirp, while cancelling out the spectral chirp introduced by CBG_1. As a result, we obtain a spatially resolved spectrum with a flat phase front.

In our setup, we used a folded configuration in which the beam is first incident obliquely on one port of the CBG, and the reflected and flipped field is then directed to the second port of the

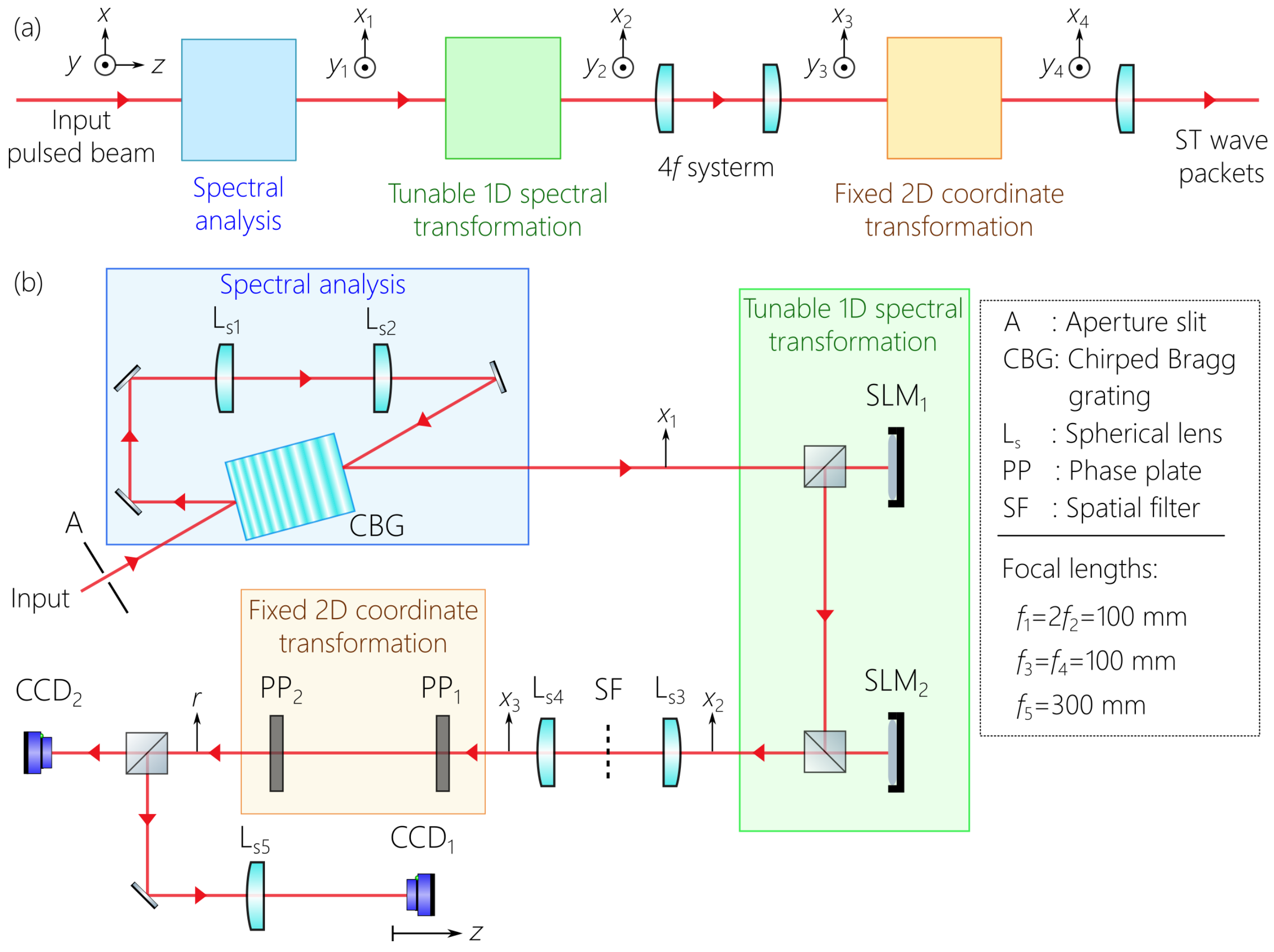

Supplementary Fig. 8. Setup for synthesizing 3D ST wave packets localized in all dimensions. (a) A conceptual layout of the setup identifying its three main stages: spectral analysis, a tunable 1D spectral transformation, and a fixed 2D coordinate transformation. (b) Detailed setup with a chirped Bragg grating (CBG) in a double-pass configuration, followed by a tunable 1D spectral transformation implemented via SLM_1 and SLM_2, and a fixed 2D coordinate transformation implemented via phase plates PP_1 and PP_2. The camera CCD_1 characterizes the 3D ST wave packets in physical space (x,y,z), and CCD_2 in the Fourier domain (k_x,k_y,λ).

same device at the same incident angle ϕ_1 [Supplementary Fig. 8(b); Spectral analysis]. Because the spatial spread of the spectrum is doubled after the CBG, we design the $4f$ imaging system between the two passes to de-magnify the field, thus matching the beam size to the CBG aperture

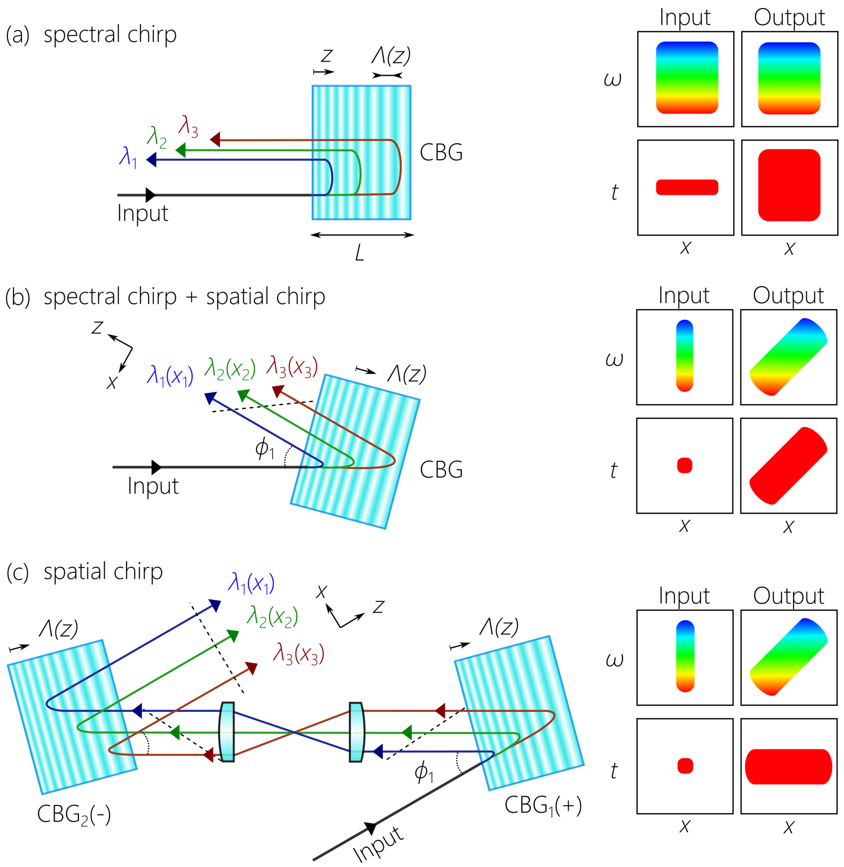

Supplementary Fig. 9. Spectral analysis via volume chirped Bragg gratings (CBGs). (a) When a plane-wave pulse is normally incident on a CBG, different wavelengths are reflected from different depths within the CBG. Spectral chirp is thus introduced into the pulse, but with no spatial chirp. (b) When a pulse is obliquely incident on the CBG, both spectral and spatial chirps are introduced. (c) By traversing a pair of identical CBGs with reversed chirp rates, the spectral chirp acquired by the plane-wave pulse from the first CBG is undone by the second, while doubling the spatial chirp. On the right-hand side in each panel, we sketch the change in the spatial distribution of the spectrum and the pulse profile before and after the CBG configuration.

size upon incidence on the second port. This double-pass configuration produces a spatially resolved spectrum with the following distribution:

$$x_1(\lambda)=\frac{1}{\beta}(\lambda-\lambda_o)\zeta(\phi_1)=\alpha(\lambda-\lambda_o), \tag{S17}$$

where $\alpha=\frac{1}{\beta}\zeta(\phi_1)$ is the linear spatial chirp rate.

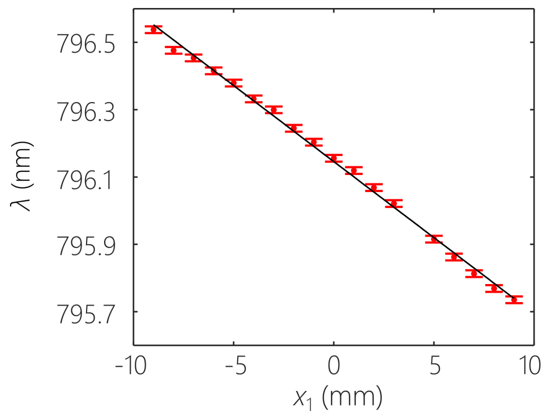

Supplementary Fig. 10. The measured spatial spread of the spectrum after the spectral analysis stage in Supplementary Fig. 8(b). The measurements confirm a linear spatial chirp over a temporal bandwidth of $\Delta\lambda\approx0.8$ nm spread over ≈18 mm in space horizontally along x_1. The straight line is a theoretical fit, and symbols are data points. Error bars correspond to the spectral resolution of the Optical Spectrum Analyzer (OSA) used in the measurements, $\delta\lambda=10$ pm.

In our experiments, we made use of a CBG (OptiGrate L1-021) with a central periodicity of $\Lambda_o=270$ nm, a chirp rate of $\beta=-30$ pm/mm, an average refractive index of $n=1.5$, and a length of $L=34$ mm. The input beam is incident at an angle $\phi_1\approx16^\circ$ with respect to the normal to the CBG entrance surface. We measure the spatially resolved spectrum by scanning a single-mode fiber (Thorlabs 780HP) connected to an optical spectrum analyzer (OSA; Advantest AQ6317B). The measured spectrum after the double-pass configuration is plotted in Supplementary Fig. 10, which verifies that the CBG produces a linear spatial chirp of rate $\alpha=-22.2$ mm/nm (from Eq. S17) centered at $\lambda_o=796.1$ nm. Therefore, at the output of the spectral-analysis stage we have a collimated, spectrally resolved optical field.

2. *Spectral uncertainty induced by CBGs*

A critical parameter characterizing ST wave packets in general is the so-called 'spectral uncertainty' $\delta\lambda$: the unavoidable 'fuzziness' in the association between spatial frequencies (k_x for 2D ST wave packets and k_r for 3D ST wave packets) and the wavelength λ. In the case of a CBG, its spectral resolving power (the smallest spectral shift that can be resolved) determines $\delta\lambda$ [22].

In the case of surface gratings, the spectral uncertainty at a central wavelength λ_{o} is given by $\delta\lambda{=}\lambda_{o}/N$, where N is the number of grating rulings covered by the incident beam. Therefore, $\delta\lambda$ here depends on the beam size and the grating period. To minimize $\delta\lambda$ of a surface grating with a fixed ruling density, it is therefore desirable to use the largest possible incident beam size. In contrast, the spectral resolving power of a CBG at normal incidence $\delta\lambda_{\text{BG}}$ – and thus the associated spectral uncertainty – is determined by its refractive-index modulation contrast and the grating length [13]. Furthermore, an additional contribution to the spectral uncertainty $\delta\lambda_{\text{GE}}$ arises at oblique incidence due to a geometric effect stemming from the finite spatial width of the input beam. When a beam of finite transverse width w is obliquely incident on the CBG, the shifted spectral intervals reflected from different points within the CBG will overlap, thereby leading to an additional contribution to the spectral uncertainty, which is proportional to the input beam width, $\delta\lambda_{\text{GE}}{\propto}w$. The total spectral uncertainty of the CBG at oblique incidence is estimated to be $\delta\lambda_{\text{CBG}}{=}\sqrt{(\delta\lambda_{\text{BG}})^{2}{+}(\delta\lambda_{\text{GE}})^{2}}$. When the beam passes through the CBG twice, a factor-of-2 *drop* is expected in the spectral uncertainty due to the doubling of the total CBG length traversed by the beam.

In our experiments, we measured $\delta\lambda_{\text{CBG}}$ as a function of the input beam width w after traversing the CBG once [Supplementary Fig. 11(a)] and twice [Supplementary Fig. 11(b)]. The beam width w is tuned using a variable-width aperture preceding the CBG [Supplementary Fig. 8(b)]. The measurements confirm that $\delta\lambda_{\text{CBG}}$ increases with w as expected. However, this trend ends when $w{\sim}2$ mm. Below this beam size, the spectral uncertainty begins to increase rather than to decrease further, thereby leading to a valley in the plots in Supplementary Fig. 11(a,b) in the vicinity of $w{\sim}2$ mm. This unexpected increase in δ_{CBG} at small w is most likely caused by diffraction inside the CBG resulting from the small input beam size. The optimum beam width is thus $w{\approx}2$ mm, which yields a minimum spectral uncertainty of $\delta\lambda_{\text{CBG}}{\approx}35$ pm in the double-pass configuration. The synthesis experiments we performed made use of $w{\approx}2$ mm, and thus we take $\delta\lambda{\geq}35$ pm for the 3D ST wave packets produced.

B. Tunable 1D spectral transformation

The second stage of the 3D ST wave-packet synthesis setup is the tunable 1D spectral transformation. This is a coordinate transformation performed along the horizontal x-axis. Because the wavelengths are arranged linearly along x after the CBG and the field is uniform along y,

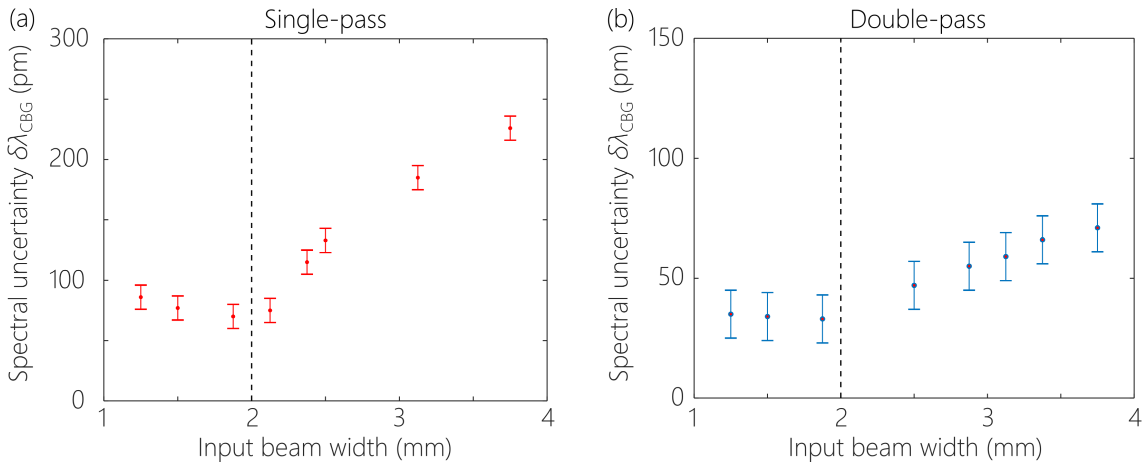

Supplementary Fig. 11. Spectral uncertainty for the CBG. (a) The measured spectral uncertainty δ_{CBG} from the CBG while varying the the input beam width in a single-pass configuration, and (b) in a double-pass configuration. The vertical dashed lines in (a) and (b) identify the operating point for our experiments. Error bars correspond to the spectral resolution of the OSA, $\delta\lambda = 10$ pm.

this 1D coordinate transformation results in a reshuffling of the arrangement of the wavelengths along x, thereby producing a spectral transformation. This spectral transformation aims at achieving two goals: (1) pre-compensating for the exponentiation in the subsequent 2D coordinate transformation; and (2) tuning the group velocity $\tilde{v}$ of the synthesized 3D ST wave packet.

The x-axis at the input plane is labelled x_1 and that at the output plane x_2. The targeted transformation then takes the form:

$$x_2 = A \ln\left(\frac{x_1}{B}\right), \tag{S18a}$$

$$y_2 = y_1, \tag{S18b}$$

where A and B are the transformation parameters, the physical significance of which will be discussed shortly. The uniform field distribution along y remains intact. This conformal mapping is implemented by means of two phase patterns placed at the input and output planes and separated by a distance d_1. The first phase distribution $\Phi_1(x_1, y_1)$ at the input plane performs the particular transformation given in Eq. S18. In other words, the wavelength λ located at position x_1 is now located at position x_2. However, such a transformation does not produce a collimated field. The second phase distribution $\Phi_2(x_2, y_2)$ placed at the output plane collimates

the transformed wave front to yield an afocal transformation. Usually, a lens is placed between the two phase plates in a $2f$-configuration. In our experiments, we do not make use of a lens and instead distribute the quadratic phase associated with such a lens between the two phase plates. The required phase profiles $\Phi_1(x_1,y_1)$ and $\Phi_2(x_2,y_2)$ – both of which are independent of y – can be derived using the methodology outlined in [15], and they take the form:

$$\Phi_1(x_1,y_1)=\frac{kA}{d_1}\left[x_1\ln\left(\frac{x_1}{B}\right)-x_1\right]-\frac{kx_1^2}{2d_1}, \tag{S19a}$$

$$\Phi_2(x_2,y_2)=\frac{kAB}{d_1}\exp\left(\frac{x_2}{A}\right)-\frac{kx_2^2}{2d_1}, \tag{S19b}$$

where $k=\frac{2\pi}{\lambda}$ is the wave number. In the setup, $\Phi_1(x_1,y_1)$ and $\Phi_2(x_2,y_2)$ are displayed on two phase-only SLMs with d_1=400 mm [Supplementary Fig. 8(b)]. Because the phase profiles according to Eq.S19 depend only on x and not y, 1D SLMs can be used here in principle. However, as we show later, utilizing 2D SLMs provides the possibility of also modulating the field along y, which translates into an azimuthal modulation after the subsequent 2D coordinate transformation.

In our experiments, we fix A=0.5 mm and tune B over the range $B=[-15,20]$ mm to control the group velocity of the 3D ST wave packets, as explained in detail later. See Supplementary Fig. 12(a) for a plot of the relationship between x_1 and x_2 when making use of these values.

The field after this 1D spectral transformation is imaged by a one-to-one $4f$ system comprising two spherical lenses L_{s3} and L_{s4}, as shown in Supplementary Fig. 8(b), from the output plane of the 1D spectral transformation (x_2,y_2) to the input plane (x_3,y_3) of the 2D coordinate transformation. Thus, the beam is flipped along the x and y axes after this $4f$ system: $x_3=-x_2$ and $y_3=-y_2$. This is important to keep in mind for determining the required transformation parameters A and B based on the desired group velocity $\widetilde{v}$. In addition, a spatial filter is placed in the Fourier plane of the $4f$ system to eliminate the undesired zeroth-order field component resulting from the limited efficiency of SLM_1 and SLM_2.

C. Fixed 2D coordinate transformation

The third stage of the spatio-temporal synthesis strategy for 3D ST wave packets is a fixed 2D coordinate transformation; see Supplementary Fig. 8(b) and Supplementary Fig. 13. This transformation performs a conformal mapping from log-polar to Cartesian coordinate systems. The input plane is spanned by Cartesian coordinates (x_3,y_3) and the output by (x_4,y_4). The

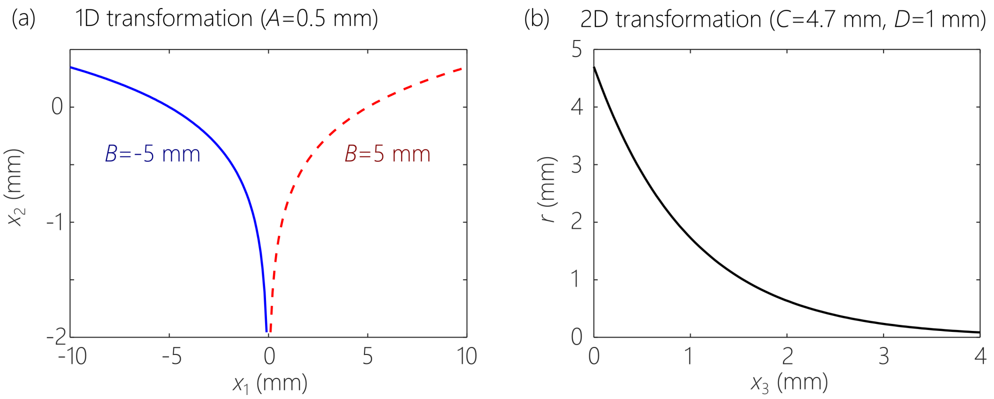

Supplementary Fig. 12. Spatial transformations associated with the 1D spectral transformation and the 2D coordinate transformation. (a) Plot of the transformation from the input plane x_1 to the output plane x_2 of the tunable 1D spectral transformation. We make use of the parameter values A=0.5 mm, and B=-5 mm (blue line) or B=5 mm (dashed red line). (b) Plot of the relationship between x_3 at the input plane to the radius r at the output plane for the fixed 2D coordinate transformation. We make use of the parameter values D=2A=1 mm and C=4.77 mm.

transformation maps the input Cartesian coordinate system (x_3,y_3) to a polar coordinate system at the output $(x_3,y_3)\rightarrow(r,\varphi)$, where $r=\sqrt{x_4^2+y_4^2}$ and $\varphi=\arctan(\frac{y_4}{x_4})$; see Supplementary Fig. 12(b). The transformation is given explicitly in the following form [14, 15, 23]:

$$r=C\exp\left(-\frac{x_3}{D}\right), \tag{S20a}$$

$$\varphi=\frac{y_3}{D}. \tag{S20b}$$

The transformation parameter D is chosen to map the vertical size of the input field $y_3=[-y_3^{\max},y_3^{\max}]$ to the range $\varphi=[-\pi,\pi]$, and thus we impose $D=\frac{y_3^{\max}}{\pi}$; the value of C is selected based on the aperture size of the optics used. The 2D coordinate transformation therefore maps a vertical line located at x_3 at the input plane into a circle of radius r at the output plane. Shifting the location of the line horizontally along x_3 at the input thus results in a radial expansion or shrinkage of the circle radius at the output according to the direction of the shift at the input [Supplementary Fig. 13(a,b)]. If the input beam is a rectangle of width Δx_3, the transformed beam is an annulus of radial thickness Δr, where the inner and outer radii of the annulus correspond to the two vertical boundaries of the input rectangle.

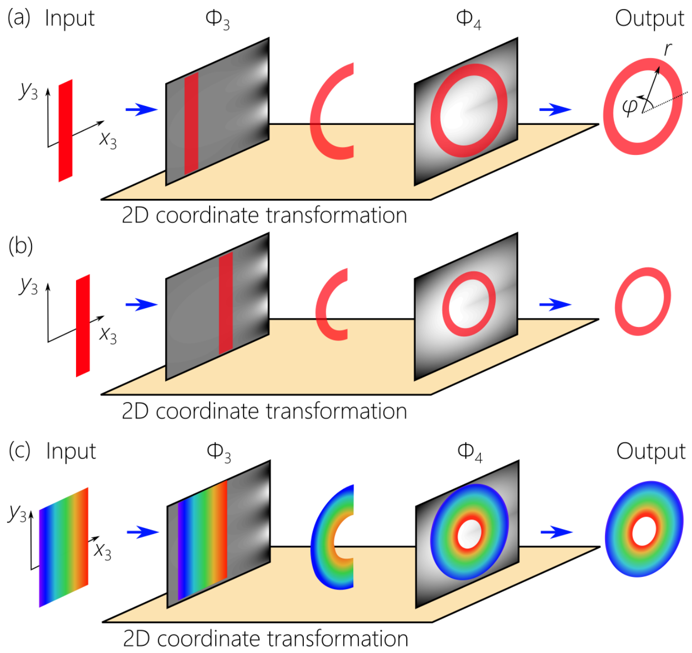

Supplementary Fig. 13. Principle of operation for the fixed 2D coordinate transformation. (a) For a monochromatic input field in the form of a vertical rectangular strip, the 2D transformation converts it into an annulus. (b) When the vertical rectangular strip is shifted horizontally, an annulus of different radius is formed at the output, which is nevertheless concentric with the annulus in (a). (c) In our experiments, the input field is a spatially extended spectrally resolved wave front. The transformation converts the input into an annulus with the wavelengths arranged radially in circles.

This 2D coordinate transformation is implemented by two 2D phase patterns (both depending on the x and y coordinates) separated by a distance d_2: Φ_3 at the input plane (x_3,y_3) and Φ_4 at the output plane (x_4,y_4). For the mapping given in Eq. S20, the phase patterns take the following form [15–17]:

$$\Phi_3(x_3,y_3)=-\frac{kCD}{d_2}\exp\left(-\frac{x_3}{D}\right)\cos\left(\frac{y_3}{D}\right)-\underbrace{\frac{k(x_3^2+y_3^2)}{2d_2}}_{\text{lens term}}, \tag{S21a}$$

$$\Phi_4(x_4,y_4)=\frac{kD}{d_2}\left[\text{atan2}(y_4,x_4)-x_4\ln\left(\frac{\sqrt{(x_4^2+y_4^2)}}{C}\right)+x_4\right]-\underbrace{\frac{k(x_4^2+y_4^2)}{2d_2}}_{\text{lens term}}, \tag{S21b}$$

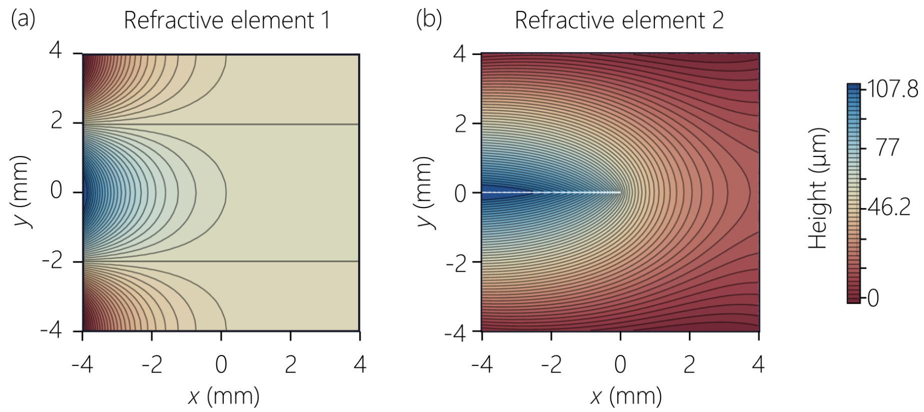

Supplementary Fig. 14. Refractive phase plates for implementing the 2D coordinate transformation. (a) Height profile of the refractive element 1, and (b) of the refractive element 2. The phase plates are designed such that the aperture size is $2y_3^{\max}=8$ mm, the distance separating them in the setup is $d_2=310$ mm, and the transformation parameters are $C=4.77$ mm, $D=1$ mm.

where $\text{atan2}(y_4,x_4)$ is 2-argument arctan function. Usually, a spherical lens is placed midway between the two phase patterns in a $2f$ configuration. Instead, the appropriate quadratic phases are added in Φ_3 and Φ_4, which are the last terms in Eq. S21(a) and Eq. S21(b) [17].

As discussed in the Methods Section in the main text, the 2D transformation was implemented using *diffractive* optics [16, 24–26] and separately *refractive* optics [17] to imprint the phase profiles in Eq. S21 via diamond-edged refractive phase plates [17] and analog diffractive phase plates [26]. The refractive optical elements used in our experiments are similar to those outlined by Lavery *et al.* in [17], in which the transformation parameters are $C=4.77$ mm, $D=\frac{3.2}{\pi}\approx 1$ mm, and $d_2=310$ mm. Each phase plate is made of the polymer PMMA (Poly methyl methacrylate) with accurately manufactured height profiles $Z_1(x_3,y_3)$ and $Z_2(x_4,y_4)$ to imprint the required phase profiles given in Eq. S21. The phase encountered by light at a wavelength λ traversing a height Z of a material of refractive index n – with respect to the phase encountered over the same distance in vacuum – is given by $\Phi=2\pi(n-1)Z/\lambda$. Thus, the height profile of the first element is $Z_1(x_3,y_3)=\frac{\lambda}{2\pi(n-1)}\Phi_3(x_3,y_3)$ [Supplementary Fig. 14(a)] and that of the profile of the second element is $Z_2(x_4,y_4)=\frac{\lambda}{2\pi(n-1)}\Phi_4(x_4,y_4)$ [Supplementary Fig. 14(b)]. See Methods in the main text for details of the fabrication and characteristics of the fabricated elements [27]. The diffractive

phase plates were fabricated in fused silica using Clemson University facilities using the process outlined in [18]. See Methods in the main text for the design parameters of the phase plates.

D. Spatial Fourier transform

After the 2D coordinate transformation, the spatially resolved wavelengths are arranged radially $r(\lambda)$. A spherical converging lens L_{s5} [Supplementary Fig. 8(b)] performs a 2D Fourier transform to produce the 3D ST wave packet in physical space. In effect, each spatial position is now mapped into a spatial frequency $k_r(\lambda){=}k\frac{r}{f}$, where f is the focal length of the lens. because of the small bandwidth $\Delta\lambda$ utilized in our experiments, we can use the approximation $k{\approx}k_o$ in the scaling of the radial spatial frequency.

E. Full system analysis

We can now combine all the stages in our synthesis system to identify the role played by the various parameters involved: the chirp rate α from the CBG; the parameters A and B from the tunable 1D spectral transformation; the parameters C and D from the fixed 2D coordinate transformation; and the focal length f of the Fourier-transforming lens. After the CBG we have $x_1(\lambda){=}\alpha(\lambda-\lambda_o)$; after the tunable 1D spectral transformation we have $x_2(\lambda){=}A\ln\{\frac{x_1(\lambda)}{B}\}$; after the $4f$ imaging system we have $x_3{=}-x_2$; after the fixed 2D coordinate transformation we have $r(\lambda){=}C\exp\{-\frac{x_3(\lambda)}{D}\}$; and, finally, after the Fourier-transforming lens we have $k_r{=}k_o\frac{r(\lambda)}{f}$. Combining all these steps, we have:

$$k_r(\lambda){=}C\frac{k_o}{f}\left(\frac{\alpha}{B}(\lambda-\lambda_o)\right)^{A/D}. \tag{S22}$$

To obtain the desired radial chirp from Eq. S15, we must impose the constraint $\frac{D}{A}{=}2$, whereupon:

$$k_r(\lambda){=}C\frac{k_o}{f}\sqrt{\frac{\alpha}{B}(\lambda-\lambda_o)}. \tag{S23}$$

The parameter $C{=}4.77$ mm is determined by the size of the optics (the aperture size is $2y_3^{\max}{=}$ 8 mm), $f{=}300$ mm, $\alpha{=}-22.2$ mm/nm is determined by the CBG, and $\lambda_o{\approx}798$ nm. This leaves B as the sole free parameter determining the chirp rate, and thus also the group velocity $\widetilde{v}$.

By comparing the result in Eq. S23 to the target spatio-temporal spectrum for 3D ST wave packets given in Eq. S15, we can obtain the group index:

$$\widetilde{n}=1+\left(\frac{C^2\alpha\lambda_o}{2f^2}\right)\frac{1}{B}=1-\frac{2.24\text{ mm}}{B} \tag{S24}$$

As a result, we can generate ST wave packets with group velocities from subluminal ($B<0$) to superluminal ($B>C^2|\alpha|\lambda_o/2f^2$), and in principle negative group velocities ($0<B<C^2|\alpha|\lambda_o/2f^2$). See Table Supplementary Table 1 for the conversion between the desired group velocity ranges and the required value of the parameter B.

F. Synthesizing pulsed Bessel beams with separable spatio-temporal spectrum

To compare the performance of 3D ST wave packets with that of the conventional pulsed Bessel beams shown in Fig. 5(a-b) in the main text, we bypass the spectral analysis and 1D spectral transformation stages and send the input laser pulses directly to the 2D transformation. Consequently, separable pulsed Bessel beams are produced. In addition, we spectrally filter $\Delta\lambda\approx0.3$ nm of the input pulses to obtain a spectral bandwidth comparable to that of the 3D ST wave packets.

	Group velocity $\widetilde{v}$	B
Positive-subluminal	$0<\widetilde{v}<c$	$B<0$
Positive-luminal	$\widetilde{v}=c$	$B=\infty$
Positive-superluminal	$c<\widetilde{v}<\infty$	$\frac{\lvert\alpha\rvert\lambda_o C^2}{2f^2}<B<\infty$
Infinite-$\widetilde{v}$	$\widetilde{v}=\infty$	$B=\frac{\lvert\alpha\rvert\lambda_o C^2}{2f^2}$
Negative-superluminal	$\widetilde{v}<-c$	$\frac{\lvert\alpha\rvert\lambda_o C^2}{4f^2}<B<\frac{\lvert\alpha\rvert\lambda_o C^2}{2f^2}$
Negative-luminal	$\widetilde{v}=-c$	$B=\frac{\lvert\alpha\rvert\lambda_o C^2}{4f^2}$
Negative-subluminal	$-c<\widetilde{v}<0$	$0<B<\frac{\lvert\alpha\rvert\lambda_o C^2}{4f^2}$

Supplementary Table 1. Conversion from the group velocity to the parameter B. This Table identifies the range taken by the values of of the parameter B (third column) corresponding to the target range of the values of the STWP group velocity $\widetilde{v}$ (second column).

Supplementary Note 3. CHARACTERIZATION OF SPACE-TIME WAVE PACKETS

We characterize the 3D ST wave packets in four domains:

1. The spatio-temporal spectral plane (k_x,k_y,λ) or (k_r,λ) to confirm the presence of desired spectral structure.

2. In physical space we follow the evolution of the time-averaged intensity $I(x,y,z)$ along the z-axis to verify the diffraction-free propagation of the 3D ST wave packets.

3. We reconstruct the spatio-temporal intensity profile $I(x,y,z;t)$ using time-resolved linear interferometry, which also enables us to estimate the group velocity $\widetilde{v}$.

4. The amplitude and phase of the complex-field envelope $\psi(x,y,z;t)=|\psi(x,y,z;t)|e^{i\phi(x,y,z;t)}$ is reconstructed using off-axis digital holography.

A. Spatio-temporal spectrum measurements

The spatio-temporal spectrum $|\widetilde{\psi}(k_x,k_y;\lambda)|^2$ is captured at CCD_2 (The ImagingSource, DMK 33UX178) as shown in Supplementary Fig. 8, which corresponds to the Fourier plane (k_x,k_y) of the synthesized 3D ST wave packets [Fig. 4(a) in the main text]. Because the camera cannot distinguish between the various wavelengths, we resolve the temporal spectrum in two steps. We first scan the fiber tip connected to an OSA along the horizontal axis x_2 after the 1D spectral transformation and determine the spatial chirp $x_2(\lambda)$. The spatial chirp after the $4f$ imaging system is identical to $x_2(\lambda)$ except for a spatial flip [Supplementary Fig. 15(a)]. In a second step, we verify experimentally the impact of the 2D coordinate transformation by scanning a vertical slit horizontally along x_3 and measure the radius of the annulus formed at the output [Supplementary Fig. 15(b)]. By combining these two measurements we obtain the spatial chirp along the radial direction $r(\lambda)$ after the 2D coordinate transformation [Supplementary Fig. 15(c)]. Finally, we obtain spatio-temporal spectrum $k_r(\lambda)$ [Supplementary Fig. 15(d) and Fig. 4(iii) in the main text] by converting from the physical space to Fourier space $k_r=k\frac{r}{f}$, where $k=\frac{2\pi}{\lambda}$ is the wave number, and $f=300$ mm is the focal lens of the Fourier-transforming lens L_{s5}. The solid curves correspond to theoretical predictions and the dots correspond to data points.

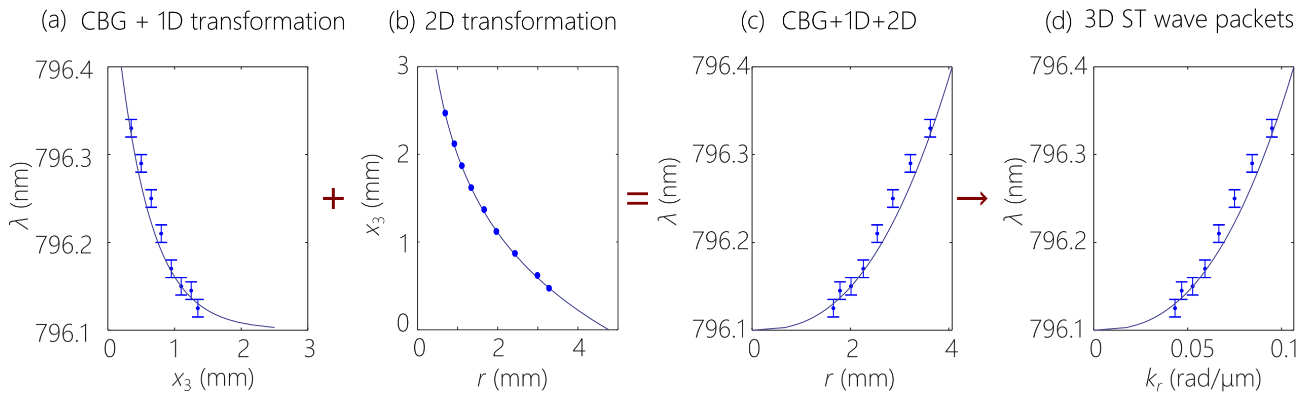

Supplementary Fig. 15. Evolution of the spatio-temporal spectrum of the 3D ST wave packets through the synthesis setup. (a) Measured relationship between λ and the transverse coordinate x_3 after the 1D spectral transformation. (b) Measured relationship between x_3 before the 2D coordinate transformation and r after it. (c) By combining (a) and (b), we obtain the relationship between λ and r after the 2D spectral transformation. (d) Transforming the relationship in (c) to one between λ and the radial spatial frequency k_r. Error bars in (a,c,d) correspond to the spectral resolution of the OSA, $\delta\lambda{=}10$ pm

B. Time-averaged intensity measurements

The axial evolution of the time-averaged intensity profile $I(x,y,z){=}\int dt|\psi(x,y,z;t)|^2$ is obtained in the physical space by scanning CCD_1 (The ImagingSource, DMK 27BUP031) along the propagation axis z [Supplementary Fig. 8(b)]. This measurement confirms the diffraction-free propagation of the 3D ST wave packets, as shown in the Fig. 5 in the main text.

C. Time-resolved intensity measurements

The spatio-temporal intensity profile of the 3D ST wave packet is measured using a Mach-Zehnder interferometer in which the spatio-temporal synthesis arrangement is placed in one arm. The second (reference) arm is traversed by the original short plane-wave laser pulses from the source (pulse width $\sim$100 fs) that encounter an optical delay line τ [Supplementary Fig. 16]. The two wave packets (the 3D ST wave packet and the reference pulse) propagate co-linearly after they are merged at the beam splitter BS_2. When the two wave packets overlap in space and

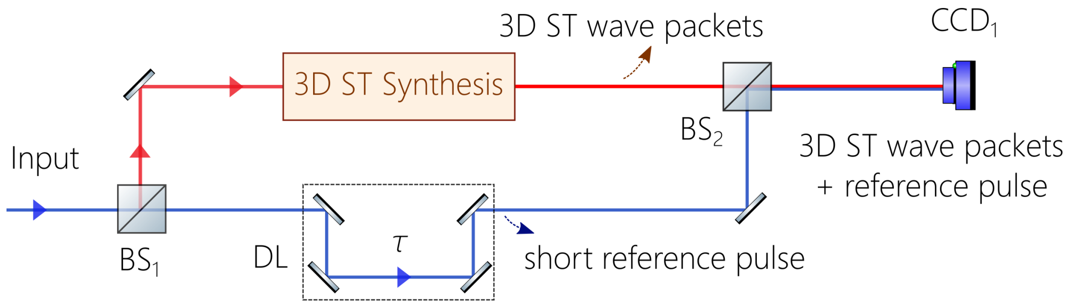

Supplementary Fig. 16. Schematic illustration of the setup for reconstructing the spatio-temporal profile of 3D ST wave packets and estimating their group velocity. The spatio-temporal synthesis arrangement from Supplementary Fig. 8(b) is placed in one arm of a Mach-Zehnder interferometer, and an optical delay τ is placed in the reference arm that is traversed by the short pulses from the initial laser source.

time, spatially resolved interference fringes are recorded by CCD_1 at a fixed axial position z, from whose visibility we extract the spatio-temporal profile at that plane. By scanning the delay τ, we can reconstruct the spatio-temporal intensity profile $I(x{=}0,y,z;\tau)$ at the fixed axial plane z. Using this approach, we obtained the wave packet profiles shown in Fig. 6(b-d) in the main text (see [3, 28] for further details).

To estimate the group velocity $\widetilde{v}$ of the 3D ST wave packet, we first arrange for the 3D ST wave packet and the reference pulse to overlap at $z{=}0$ as described above. We then axially displace CCD_1 to a different axial position Δz, which results in a loss of interference visibility due to the group-velocity mismatch between the 3D ST wave packet traveling at $\widetilde{v}{=}c\tan\theta$ (where θ is the spectral tilt angle) and the reference pulse traveling at $\widetilde{v}{=}c$. The interference is retrieved, however, by adjusting the delay by Δt in the reference arm, from which we obtain the relative group delay between the two wave packets and thence the group velocity $\widetilde{v}{=}\frac{\Delta z}{\Delta t}$ for the 3D ST wave packet [Fig. 6(e) in the main text]. By repeating this procedure for ST wave packets with different θ (by varying the parameter B), we obtain the data plotted in Fig. 6(f) in the main text.

The uncertainty in the group-velocity measurement is estimated using the propagation-of-errors principle [29]. In our case, the largest contribution to errors in estimating $\widetilde{v}$ stems from the uncertainty δt in estimating the group delay Δt, which is limited by the pulse-width ΔT,

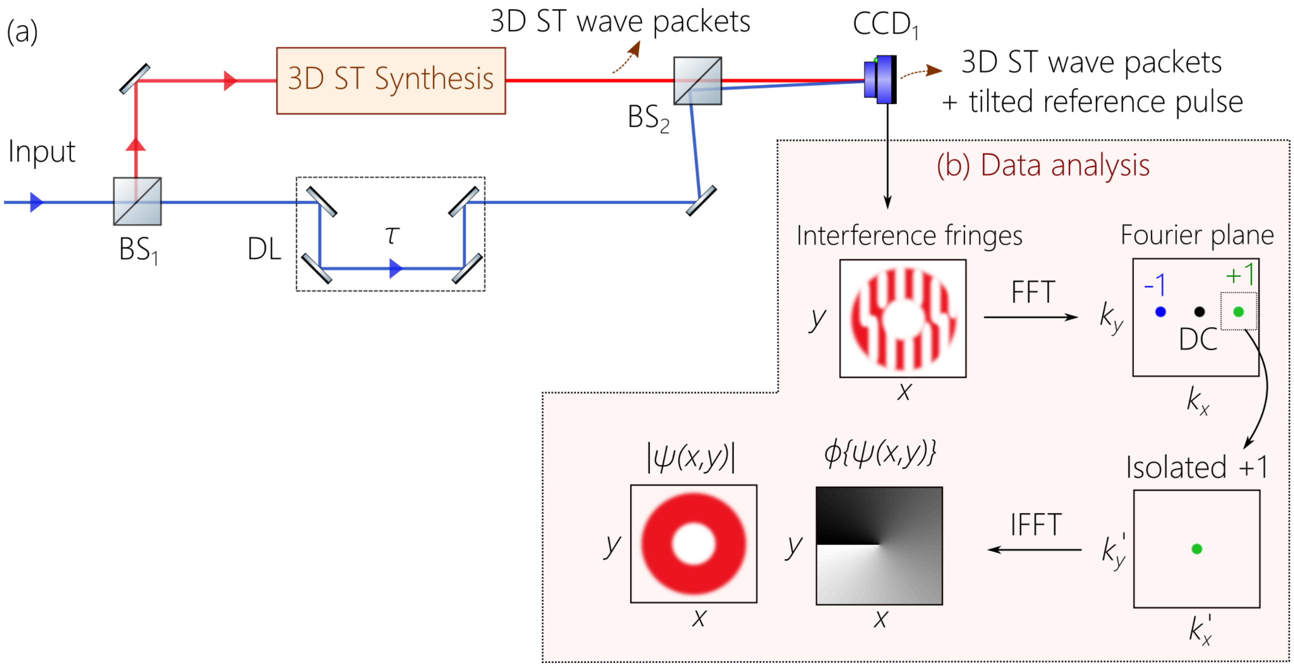

Supplementary Fig. 17. Schematic depiction of the off-axis holography strategy for reconstructing the complex field for 3D ST wave packets. (a) The system is similar to that in Supplementary Fig. 16 except that the reference pulse is spatially tilted with respect to the 3D ST wave packet. (b) Outline of the procedure for estimating the field amplitude and phase from the spatially resolved interference fringes.

which we set at $\delta t{=}\Delta T/10$. The error in the estimated value of $\widetilde{v}$ is $\delta\widetilde{v}{=}|\frac{\partial\widetilde{v}}{\partial t}|\delta t{=}\frac{\widetilde{v}^2}{\Delta z}\delta t$. Using this relationship, we calculate the error bars in Fig. 6(f) in the main text.

D. Measurements of the field amplitude and phase for 3D ST wave packets

Finally, we make use of off-axis digital holography [30–32] to obtain the field amplitude and phase of 3D ST wave packets at fixed locations along z and at fixed instances in time τ. This is especially crucial to measure the phase of the OAM-carrying 3D ST wave packets shown in Fig. 7(b) in the main text.

We make use of the off-axis digital holography (ODH) methodology [30–32] to reconstruct the complex field of 3D ST wave packets $\psi(x,y,z;t){=}|\psi(x,y,z;t)|e^{i\phi(x,y,z;t)}$. For this purpose, the same Mach-Zehnder configuration from the previous section is exploited but with a slight modification

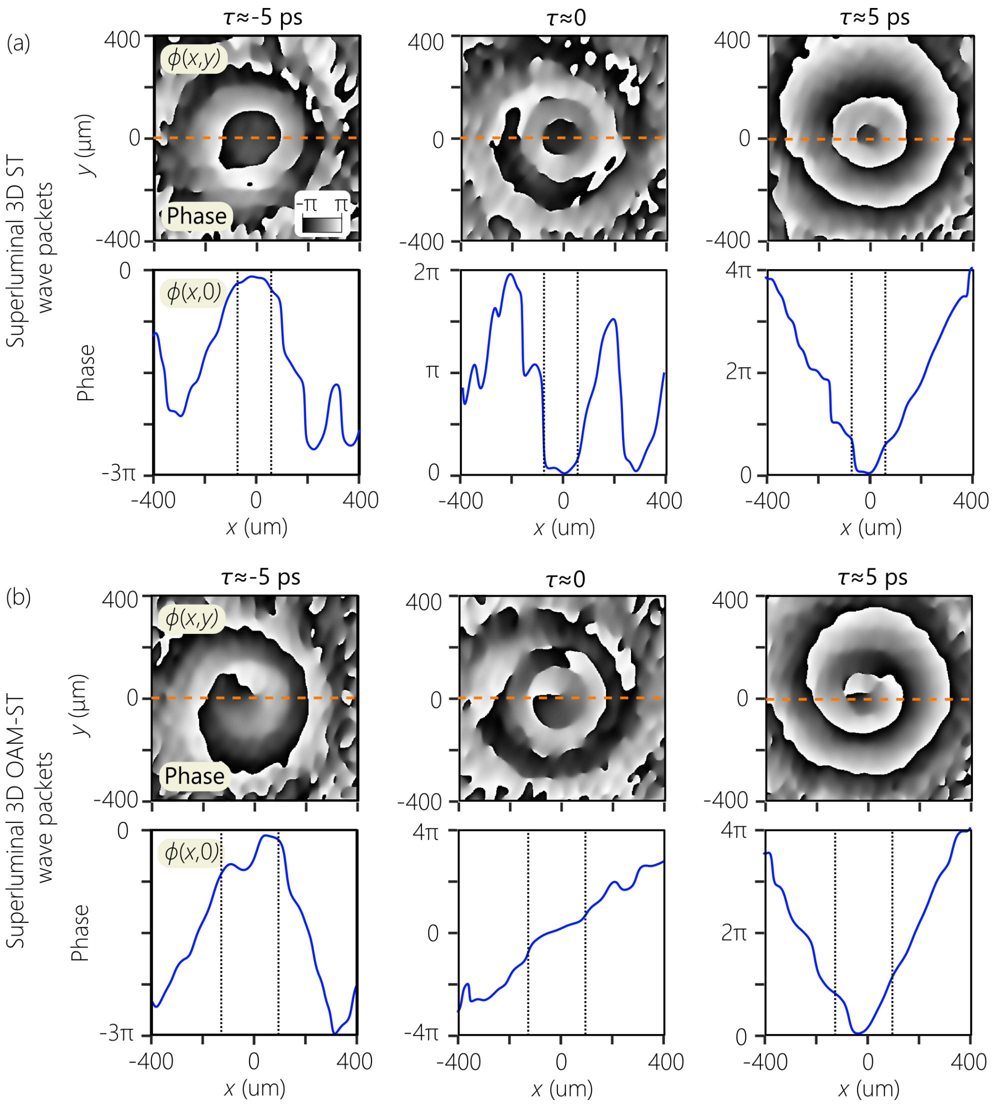

Supplementary Fig. 18. Measured phase profiles for 3D ST wave packets with and without OAM. (a) Measured transverse phase profile $\phi(x,y)$ (first row) and on-axis phase profile $\phi(x,y\!=\!0)$ along the x-axis (second row) at delays $\tau\approx0,\pm5$ ps for a superluminal 3D ST wave packet. Here $z\!=\!30$ mm and $\ell\!=\!0$; i.e., there is no OAM structure. (b) Same as (a) except that $\ell\!=\!1$; i.e., OAM structure has been introduced into the field.

– we add a small angle between the propagation directions of the short reference pulse and the 3D ST wave packets, which are arranged to overlap in space and time at a fixed axial position z [Supplementary Fig. 17(a)]. The interference pattern captured on CCD_1 contains a constant background term and an interference term of interest [Supplementary Fig. 17(b)]. Following

the ODH algorithm, we perform a digital fast Fourier transform (FFT) to separate the constant background from the interference term. By digitally isolating the first diffraction order of the Fourier-transformed image, we access the interference term that contains the complex field. Finally, the inverse FFT of the centered first term gives the amplitude $|\psi(x,y,z;\tau)|$ and phase $\phi\{\psi(x,y,z;\tau)\}$ of the 3D ST wave packets at that axial location z and time τ (see [30–32] for more details). By repeating this procedure for several instances τ we obtain the data plotted in Fig. 7 of the Main text.

The measured phase structure is plotted in Supplementary Fig. 18(a) for ℓ=0 and in Supplementary Fig. 18(b) for ℓ=. In the first case, in absence of OAM, the phase distribution takes a Gaussian form. The curvature increases as we move away from the center of the wave packet τ=0. We highlight the phase along the center of the wave front y=0, and especially where the intensity of the field is appreciable (between the two vertical dotted lines). Here the phase is flat at τ=0, and becomes Gaussian as τ increases. This is to be expected for a monochromatic Gaussian beam going through its focal point (where the phase front is flat). However, this is seen here in the time domain, which is a manifestation of so-called 'time diffraction' [33–35].

In presence of non-zero OAM, superimposed on the above-described behavior of the phase front is a helical phase distribution. At τ=0 the helical phase front flattens out, but appears as we move away from the wave packet center [Supplementary Fig. 18(b)]. Once again, this is the expected behavior in space for a diffracting OAM mode when examined from the beam waist outward, but observed here in the time domain.

SUPPLEMENTARY REFERENCES

[1] H. E. Kondakci and A. F. Abouraddy, "Diffraction-free pulsed optical beams via space-time correlations," Opt. Express **24**, 28659–28668 (2016).

[2] H. E. Kondakci and A. F. Abouraddy, "Diffraction-free space-time beams," Nat. Photon. **11**, 733–740 (2017).

[3] H. E. Kondakci and A. F. Abouraddy, "Optical space-time wave packets of arbitrary group velocity in free space," Nat. Commun. **10**, 929 (2019).

[4] M. Yessenov, B. Bhaduri, H. E. Kondakci, and A. F. Abouraddy, "Classification of propagation-invariant space-time light-sheets in free space: Theory and experiments," Phys. Rev. A **99**, 023856 (2019).

[5] M. Yessenov, B. Bhaduri, L. Mach, D. Mardani, H. E. Kondakci, M. A. Alonso, G. A. Atia, and A. F. Abouraddy, "What is the maximum differential group delay achievable by a space-time wave packet in free space?" Opt. Express **27**, 12443–12457 (2019).

[6] M. Yessenov, B. Bhaduri, H. E. Kondakci, and A. F. Abouraddy, "Weaving the rainbow: Space-time optical wave packets," Opt. Photon. News **30**, 34–41 (2019).

[7] A. M. Shaarawi and I. M. Besieris, "Relativistic causality and superluminal signalling using X-shaped localized waves," J. Phys. A **33**, 7255–7263 (2000).

[8] J. Durnin, J. J. Miceli, and J. H. Eberly, "Diffraction-free beams," Phys. Rev. Lett. **58**, 1499–1501 (1987).

[9] J. Turunen and A. T. Friberg, "Propagation-invariant optical fields," Prog. Opt. **54**, 1–88 (2010).

[10] H. E. Hernández-Figueroa, E. Recami, and M. Zamboni-Rached, eds., *Non-diffracting Waves* (Wiley-VCH, 2014).

[11] K. J. Parker and M. A. Alonso, "The longitudinal iso-phase condition and needle pulses," Opt. Express **24**, 28669–28677 (2016).

[12] S. Kaim, S. Mokhov, B. Y. Zeldovich, and L. B. Glebov, "Stretching and compressing of short laser pulses by chirped volume Bragg gratings: analytic and numerical modeling," Opt. Eng. **53**, 051509 (2014).

[13] L. B. Glebov, V. Smirnov, R. Eugeniu, I. Cohanoschi, L. Glebova, O. V. Smolski, J. Lumeau, C. Lantigua, and A. Glebov, "Volume-chirped Bragg gratings: monolithic components for stretching and

compression of ultrashort laser pulses," Opt. Eng. **53**, 051514 (2014).

[14] O. Bryngdahl, "Geometrical transformations in optics," J. Opt. Soc. Am. **64**, 1092–1099 (1974).

[15] W. J. Hossack, A. M. Darling, and A. Dahdouh, "Coordinate transformations with multiple computer-generated optical elements," J. Mod. Opt. **34**, 1235–1250 (1987).

[16] G. C. G Berkhout, M. P. J. Lavery, J. Courtial, M. W. Beijersbergen, and M. J. Padgett, "Efficient sorting of orbital angular momentum states of light," Phys. Rev. Let. **105**, 153601 (2010).

[17] M. P. J. Lavery, D. J. Robertson, G. C. G Berkhout, G. D. Love, M. J. Padgett, and J. Courtial, "Refractive elements for the measurement of the orbital angular momentum of a single photon," Opt. Express **20**, 2110–2115 (2012).

[18] J. W. Sung, H. Hockel, J. D. Brown, and E. G. Johnson, "Development of a two-dimensional phase-grating mask for fabrication of an analog-resist profile," Appl. Opt. **45**, 33–43 (2006).

[19] B. E. A. Saleh and M. C. Teich, *Fundamentals of Photonics* (Wiley, 2019).

[20] K. Liao, M. Cheng, E. Flecher, V. I. Smirnov, L. B. Glebov, and A. Galvanauskas, "Large-aperture chirped volume Bragg grating based fiber CPA system," Opt. Express **15**, 4876–4882 (2007).

[21] R. Sun, D. Jin, F. Tan, S. Wei, C. Hong, J. Xu, J. Liu, and P. Wang, "High-power all-fiber femtosecond chirped pulse amplification based on dispersive wave and chirped-volume Bragg grating," Opt. Express **24**, 22806–22812 (2016).

[22] E. G. Loewen and E. Popov, *Diffraction gratings and applications* (CRC Press, 2018).

[23] Y. Saito, S. Komatsu, and H. Ohzu, "Scale and rotation invariant real time optical correlator using computer generated hologram," Opt. Commun. **47**, 8–11 (1983).

[24] M. P. J. Lavery, G. C. G. Berkhout, J. Courtial, and M. J. Padgett, "Measurement of the light orbital angular momentum spectrum using an optical geometric transformation," J. Opt. **13**, 064006 (2011).

[25] G. C. G. Berkhout, M. P. J. Lavery, M. J. Padgett, and M. W. Beijersbergen, "Measuring orbital angular momentum superpositions of light by mode transformation," Opt. Lett. **36**, 1863–1865 (2011).

[26] W. Li, K. S. Morgan, Y. Li, J. K. Miller, G. White, R. J. Watkins, and E. G. Johnson, "Rapidly tunable orbital angular momentum (OAM) system for higher order Bessel beams integrated in time (HOBBIT)," Opt. Express **27**, 3920–3934 (2019).

[27] T. A. Dow, M. H. Miller, and P. J. Falter, "Application of a fast tool servo for diamond turning of non-rotationally symmetric surfaces," Precis. Eng. **13**, 243–250 (1991).

[28] B. Bhaduri, M. Yessenov, and A. F Abouraddy, "Anomalous refraction of optical spacetime wave packets," Nat. Photon. **14**, 416–421 (2020).

[29] P. R. Bevington and D. K. Robinson, *Data Reduction and Error Analysis for the Physical Sciences* (McGraw-Hill, New York, 2002).

[30] E. Cuche, F. Bevilacqua, and C. Depeursinge, "Digital holography for quantitative phase-contrast imaging," Opt. Lett. **24**, 291–293 (1999).

[31] E. Cuche, P. Marquet, and C. Depeursinge, "Spatial filtering for zero-order and twin-image elimination in digital off-axis holography," Appl. Opt. **39**, 4070–4075 (2000).

[32] E. Sánchez-Ortiga, A. Doblas, G. Saavedra, M. Martínez-Corral, and J. Garcia-Sucerquia, "Off-axis digital holographic microscopy: practical design parameters for operating at diffraction limit," Appl. Opt. **53**, 2058–2066 (2014).

[33] M. A. Porras, "Gaussian beams diffracting in time," Opt. Lett. **42**, 4679–4682 (2017).

[34] H. E. Kondakci and A. F. Abouraddy, "Airy wavepackets accelerating in space-time," Phys. Rev. Lett. **120**, 163901 (2018).

[35] M. Yessenov, L. A. Hall, S. A. Ponomarenko, and A. F. Abouraddy, "Veiled Talbot effect," Phys. Rev. Lett. **125**, 243901 (2020).
